# Burden and risk factors for gallbladder and biliary tract diseases in China from 1990 to 2021 and burden predictions of risk factors for the next 15 years

**DOI:** 10.3389/fmed.2025.1528608

**Published:** 2025-08-15

**Authors:** Shijun Zhao, Yan Zhou, Wangyou Tang, Cheng Zhao, Lang Wang, Xianglin Zhu, Hao Liang, Jie Zhang, Tian Gao, Yinlu Ding

**Affiliations:** ^1^Department of General Surgery, The Second Hospital of Shandong University, Jinan, Shandong, China; ^2^Anqiu Municipal Hospital, Weifang, Shandong, China

**Keywords:** gallbladder and biliary tract diseases, Global Burden of Disease Study, body mass index, China, prediction

## Abstract

**Background:**

Gallbladder and biliary tract diseases (GBDT) is a common digestive disorder; however, comprehensive epidemiological data from China are still limited.

**Methods:**

This study utilized the Global Burden of Disease Study 2021 database to examine the burden of GBDT from 1990 to 2021. The key metrics analyzed included age-standardized incidence rate (ASIR), prevalence rate (ASPR), rate of mortality (ASMR), and age-standardized disability-adjusted life years rate (ASDR). Furthermore, the study analyzed trends and future projections concerning GBDT linked to high body mass index (HBMI). Joinpoint regression analyses were performed to evaluate trends in disease burden from 1990 to 2021. Furthermore, the study analyzed trends in mortality and DALYs attributable to GBDT linked to high HBMI and made projections for HBMI-related GBDT mortality and disability-adjusted life years (DALYs) over the next 15 years.

**Results:**

From 1990 to 2021, the ASPR and ASIR of GBDT in China showed fluctuating trends, and the ASPR and ASIR of GBDT in globally also fluctuated similarly, but with a smaller amplitude, and the overall trend showed a decreasing trend. The ASMR showed a lower level with a stable trend, and the ASDR also showed a lower level but slowly decreased annually. The incidence, prevalence, mortality and DALYs of GBDT were correlated with old age and female. The burden of disease attributable to GBDT due to HBMI was concentrated in the very old. The BAPC model predicts an increase in the DALYs and a decrease in mortality, deaths, and DALYs rate for GBDT attributable to HBMI over the next 15 years.

**Conclusion:**

GBDT remains a major global public health problem, and the development trend of GBDT in China has far-reaching global implications. In China, the number of incident cases of GBDT and ASIR increased between 1990 and 2021, and GBDT, due to HBMI, poses a greater disease burden in China, both in the past and in the future.

## 1 Introduction

GBDT, which include conditions such as cholelithiasis and cholecystitis, represent significant gastrointestinal disorders that frequently necessitate hospital admissions. Although these conditions typically have a low mortality rate, they often require surgical intervention, positioning them as a leading cause of healthcare expenditure in the United States, with annual costs reaching $16.9 billion (1)—representing a 191.4% increase from $5.8 billion in 1998 (2)—and contributing substantially to global health and economic burdens. Cholelithiasis, the most common form of GBDT, has prevalence rate of 10–15% for gallstones and 10–20% for choledocholithiasis in adults (3). Despite its increasing prevalence and considerable burden (4), approximately 70% of individuals with cholelithiasis remain asymptomatic, although 10–20% may develop symptoms over a decade (5). Asymptomatic gallstones can potentially lead to severe conditions such as acute or chronic cholecystitis, cholangitis, pancreatitis, and even cancers of the liver, biliary tract, and pancreas due to inflammation, altered bile flow, and hormonal changes (6).

In China, the prevalence of cholelithiasis is 11.0% (7). Gallstone-related cystic duct obstruction accounts for 90–95% of acute cholecystitis cases, whereas 5–10% of patients with acute cholecystitis experience non-calculous cholecystitis (8). Noncalculous cholecystitis poses greater risks of gallbladder perforation and complications than does calculous cholecystitis (9).

Overweight and obesity are significant public health concerns globally, and the number of overweight and obese adults could reach 1.35 billion and 573 million, respectively, by 2030 (10). Specifically, in China, these numbers are projected to reach 540 million overweight and 150 million obese adults (11). By 2035, the worldwide economic impact of overweight and obesity could reach $4.32 trillion, nearly 3% of the world’s GDP, creating a financial burden equivalent to the impact of the COVID-19 pandemic in 2020 (12). HBMI criteria, which are based on mixed-effects modeling and consider factors such as country, year, age, and sex (13), define obesity in China as a BMI over 28 kg/m^2^ and are associated with an increased risk of gallbladder diseases (14, 15).

Currently, GBD-based reports on GBDT focus on global and regional macro assessments, identifying trends and risks at these levels from 1990 to 2021 (16, 17). However, a deeper exploration of the variations between countries and regions is often overlooked, and specific national crude incidence rate circumstances are neglected. Given China’s position as the world’s second most populous country, understanding its GBDT burden is crucial for the global context. Using up-to-date GBD data, this study investigates China’s GBDT status and trends from 1990 to 2021, focusing on the gender- and age-related burdens linked to HBMI, and projects the future burden attributable to high BMI over the next 15 years. This comprehensive analysis supports healthcare planning, health promotion, and effective resource allocation.

## 2 Methods

### 2.1 Data

The Global Burden of Disease, Injury, and Risk Factor Study is managed by the Institute for Health Metrics and Evaluation at the University of Washington. This essential resource analyzes global health challenges and offers in-depth insights into 371 diseases and 88 risk factors across 204 countries and territories from 1990 to 2021 (18, 19). Available data can be accessed through the Global Health Data Exchange platform,^1^ enabling researchers to track health trends, evaluate risk factors, and pinpoint critical areas for health interventions. The methodologies and statistical models used in the GBD are thoroughly detailed in the scholarly literature to ensure transparency and facilitate replication (18, 19).

In this study, we focus on Chinese and global GBD data related to GBDT from 1990 to 2021. GBDT, as categorized by the GBD, includes conditions such as gallstones, cholecystitis, and cholangitis while excluding cancers of the gallbladder and biliary tract. The relevant ICD-10 codes for these diseases are K80-K83.9 (20). The importance of this dataset lies in its capacity to offer a detailed overview of disease prevalence, inform public health strategies, and guide resource allocation without requiring ethical approval or patient consent, as it does not contain identifiable personal information.

### 2.2 Risk factors

The 2021 GBD study utilized a comparative risk assessment framework to compare mortality and DALYs associated with 87 different risk factors and combinations of these factors at different levels, countries, and regions around the world (18). These risk factors were grouped into three broad categories: environmental, metabolic, and behavioral. Among the risk factors assessed in this study, HBMI was considered the most critical risk factor for GBDT.

### 2.3 Disease burden

In this study, prevalence, morbidity, mortality, and DALYs were utilized to evaluate the disease burden associated with GBDT. DALYs serve as a standardized measure to quantify the disease burden, representing the total years of healthy life lost due to the disease, which includes both years of life lost (YLLs) and years lived with disability (YLDs). This value is calculated via the following formula:


DALYs=YLLs+YLDs

### 2.4 Statistical analysis

From 1980 to 2021, the GBD database utilized age-standardized rate (ASR) population estimates to represent the average global population while eliminating the influence of population size changes over time. The ASR is computed via the following equation (21):


$$\mathrm{ASR}=\frac{\sum_{i=1}^{A}a_{i}w_{i}}{\sum_{i=1}^{A}w_{i}}\times 100,000$$

To describe the disease burden for GBDT from 1990 to 2021, ASIRs, ASPRs, ASDRs, and DALYs were utilized. The central estimate for each variable was calculated by averaging all sampled values, while the 95% uncertainty intervals (UIs) were derived from the 2.5th and 97.5th percentile value (22). Data visualization included heatmaps and bubble plots to illustrate trends in the burden of disease attributed to HBMI, categorized by sex throughout this period.

To analyze trends in disease burden, the mean annual percentage change (AAPC) along with the associated 95% confidence intervals (95% CI) were calculated via the joinpoint software developed by the National Cancer Institute in Rockville, MD, USA (23).

To estimate the burden of GBDT in China from 2022 to 2036, a BAPC analysis was conducted. This analysis relied on mortality data and DALYs from the GBD database, which covers the period from 1990 to 2021. The population size for 2036 was estimated via standardized population figures from the 2017 GBD database. The BAPC model is founded on the Age-Period-Cohort (APC) framework, which is a commonly applied method for analyzing trends in morbidity and mortality associated with chronic diseases. In our BAPC model implemented using the BAPC package (v0.0.36) in R software, we employed default prior settings consistent with established practices in epidemiological forecasting: a second-order random walk prior (RW2) with its precision parameter assigned an inverse gamma distribution. This prior specification controls smoothness to prevent overfitting, and its validity has been previously described (24). By addressing the linear relationships among age, period, and cohort inherent in traditional APC models, BAPC enhances the standard model by incorporating prior information about unknown parameters and using sample data to generate posterior distributions. The non-identifiability issue inherent in the APC model was resolved through the linear extrapolation mechanism of the RW2 prior. The RW2 prior assumes similarity between adjacent effects and extrapolates future values via their conditional distribution. This approach ensures the identifiability of the APC model. Within this Bayesian framework, the integrated nested Laplace approximation algorithm is frequently utilized to estimate models that provide direct approximations of the posterior marginal distribution. The prediction process typically involves the use of the BAPC and INLA packages in the R programming environment (25).

## 3 Results

### 3.1 Description of the burdens of GBDT in China and globally

#### 3.1.1 Incidence of GBDT in China and globally

In China, the number of incidence cases of GBDT has increased dramatically, from 1,261,128 cases (95% CI: 1,074,443–1,486,844) in 1990 to 24,043,973 cases (95% CI: 20,428,128–28,111,022) in 2021, representing a cumulative growth of 90.66%. Comparatively, the global incidence of GBDT rose from 45,747,542 cases in 1990 (95% CI: 39,294,104–53,777,201) to 73,246,893 cases in 2021 (95% CI: 63,105,084–84,905,970), resulting in a cumulative increase of 60.11%. However, the global ASIR decreased from 992.88 cases per 100,000 people in 1990 (95% CI: 854–1,165.06) to 865.4 cases per 100,000 people in 2021 (95% CI: 747.64–1,000.77). In China, the ASIR exhibited a different trend, increasing from 1,202.91 cases per 100,000 people in 1990 (95% CI: 1,028.36-1,419.64) to 1,296.12 cases per 100,000 people in 2021 (95% CI: 1,114.27–1,496.44). Moreover, the incidence of AAPC in China from 1990to 2021 increased by 0.26% (95% CI: 0.23–0.29), whereas the global AAPC decreased by 0.45% (95% CI: −0.46–0.44) during the same period (Table 1).

**Table 1:** Number of cases, age-standardized incidence rate, prevalence rate, mortality rate, and DALYs rate for China and globally in 1990 and 2021, along with the corresponding AAPCs.

Location	Measure	1990 All-ages cases *n* (95% UIs)	ASR per 100,000 people *n* (95% UIs)	2021 All-ages cases *n* (95% UIs)	ASR per 100,000 people *n* (95% UIs)	1990–2021 AAPC *n* (95% CI)
China	Incidence	12,611,128 (10,742,443–14,886,844)	1,202.91 (1028.36–1,419.64)	24,043,973 (20,428,128–28,111,022)	1,296.12 (1114.27–1,496.44)	0.26 (0.23–0.29)
	Prevalence	41,817,141 (35,643,523–49,931,025)	4,350.36 (3,710.28–5,154.29)	82,838,171 (69,665,692–97,028,157)	4,305.47 (3706.43–5,029.01)	−0.03 (−0.08–0.02)
	Deaths	16,634 (8,709–21,420)	2.99 (1.59–3.86)	20,858 (15,356–32,179)	1.2 (0.89–1.84)	−2.99 (−3.19– –2.78)
	DALYs	1,294,556 (914,235–1,753,211)	145.35 (102.9–195.13)	2,096,710 (1,439,683–2,981,286)	109.61 (75.98–155.14)	−0.92 (−0.98– –0.86)
Global	Incidence	45,747,542 (39,294,104–53,777,201)	992.88 (854–1,165.06)	73,246,893 (63,105,084–84,905,970)	865.4 (747.64–1,000.77)	−0.45 (−0.46– –0.44)
	Prevalence	150,370,152 (129,256,828–77,790,740)	3,422.23 (2,914.36–4,015.23)	251,574,857 (215,272,108–93,541,994)	2,966.67 (2,551.75–3,447.62)	−0.45 (−0.48– –0.42)
	Deaths	76,357 (60,902–85,367)	2.25 (1.82–2.51)	131,110 (113,367–155,750)	1.62 (1.4–1.92)	−1.04 (−1.15– –0.94)
	DALYs	4,929,503 (3,734,854–6,474,067)	116.09 (89.07–150.8)	7,734,159 (5,859,796–10,306,276)	91.73 (69.6–122.11)	−0.76 (−0.83– −0.70)

ASR, Age-standardized rate; DALYs, Disability-adjusted life-years; AAPC, Average Annual Percentage Change; UIs, Uncertainty intervals.

#### 3.1.2 Prevalence of GBDT in China and globally

The number of prevalent cases of GBDT has significantly changed over the past few decades. In China, prevalent cases increased from approximately 41.8 million in 1990 to approximately 82.8 million in 2021, reflecting a cumulative growth of 98.1%. Similarly, global prevalent cases rose from approximately 150.4 million in 1990 to approximately 251.6 million in 2021, representing a cumulative increase of 67.3%. When the ASPR was examined, the global rate per 100,000 people declined from 3,422.23 in 1990 to 2,966.67 in 2021. In contrast, China’s ASPR slightly decreased from 4,350.36 to 4,305.47 during the same period. From 1990 to 2021, the AAPC in global prevalence showed a modest decline of 0.45%. However, in China, the AAPC showed only a marginal decrease of 0.03% (Table 1).

#### 3.1.3 Deaths of GBDT in China and globally

In 2021, GBDT resulted in 131,110 deaths globally (95% CI: 113,367–155,750), reflecting a 71.71% increase since 1990. In China, the ASMRs of these diseases increased by 25.39% during the same period. The ASMR decreased from 2.25 deaths per 100,000 people in 1990 (95% CI: 1.82–2.51) to 1.62 per 100,000 in 2021 (95% CI: 1.40–1.92). In China, the ASMR also decreased from 2.99 deaths per 100,000 (95% CI: 1.59–3.86) in 1990 to 1.20 deaths per 100,000 (95% CI: 0.89–1.84) in 2021. Furthermore, the AAPC of global mortality decreased by 1.04% (95% CI: −1.15-0.94) from 1990 to 2021, whereas China’s AAPC declined by 2.99% (95% CI: −3.19–2.78) during the same timeframe (Table 1).

#### 3.1.4 DALYs of GBDT in China and globally

Globally, the number of DALYs attributed to GBDT rose from 4,929,503 in 1990 (95% CI: 3,734,854–6,474,067) to 7,734,159 in 2021 (95% CI: 5,859,796–10,306,276), indicating a 56.90% increase. In China, DALYs associated with GBDT increased by 61.96% between 1990 and 2021. The ASDR decreased from 116.09 per 100,000 people in 1990 (95% CI: 89.07–150.8) to 91.73 per 100,000 in 2021 (95% CI: 69.6–122.11). Similarly, in China, the ASDR declined from 145.35 per 100,000 (95% CI: 102.9–195.13) in 1990 to 109.61 per 100,000 (95% CI: 75.98–155.14) in 2021. The AAPC of DALYs globally decreased by 0.76% (95% CI: −0.83-0.70) from 1990 to 2021, whereas in China, it declined by 0.92% (95% CI: −0.98–0.86) during the same period (Table 1).

### 3.2 Global and Chinese trends in the GBDT disease burden

**Figure 1:**
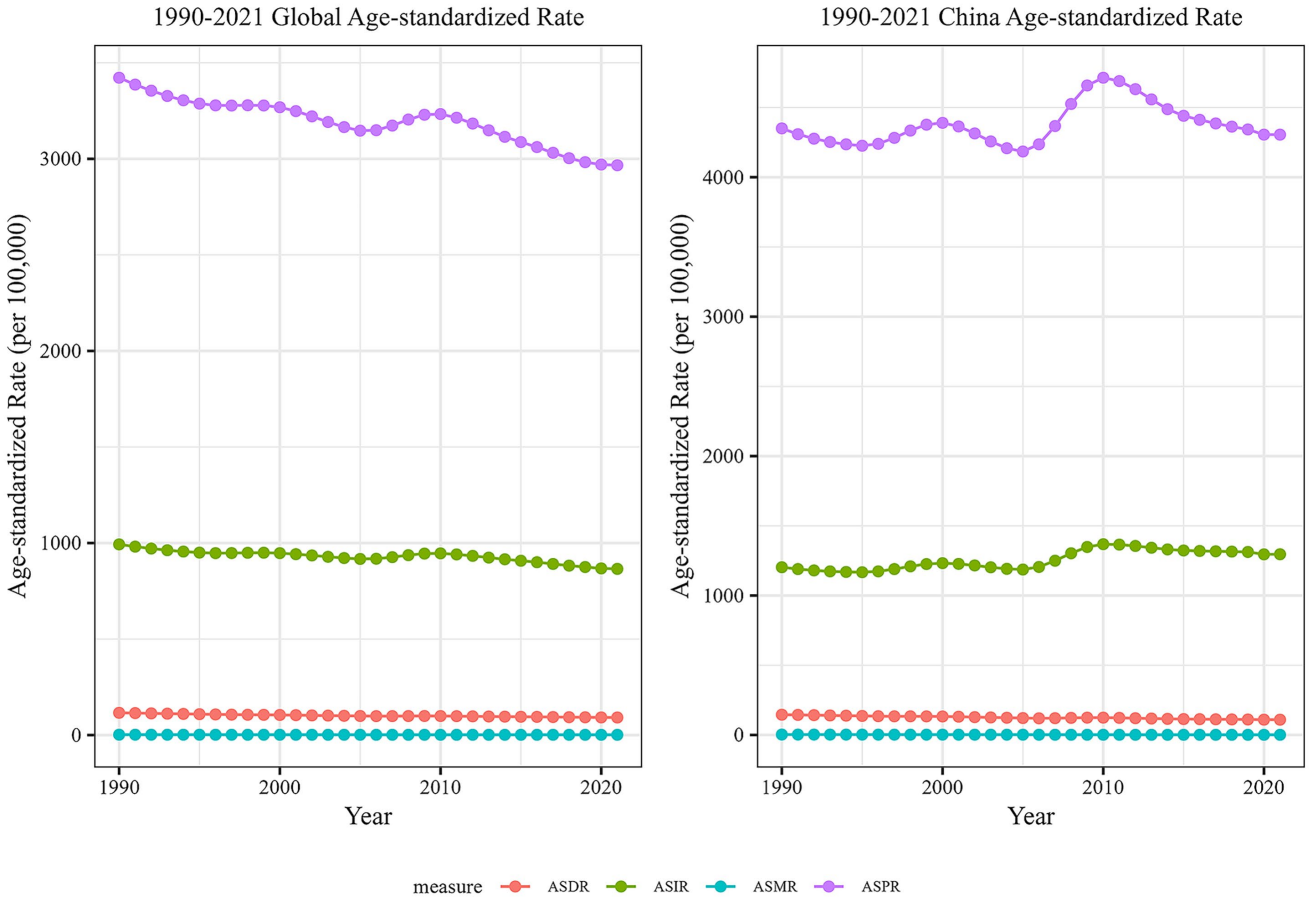
Comparison of trends in ASIR, ASPR, ASMR, and ASDR for GBDT in China and global from 1990 to 2021.

Between 1990 and 2021, the ASPR of GBDT in China displayed a fluctuating pattern. It initially declined slowly from 1990 to 1995, followed by a gradual increase after 1995. The rate decreased again in 2000 and then significantly increased from 2005 to 2010, exceeding the levels recorded in 1990. By 2021, the ASPR had slightly decreased compared with its 1990 values. In contrast, the global ASPR for GBDT steadily declined from 1990 to 2000, remained stable during the next phase, and continued to decrease after 2000, with a modest increase between 2005 and 2010 before showing an overall downward trend. The ASIR for GBDT in China also exhibited an overall slow fluctuation, mirroring the trends observed in the ASPR. By 2021, the ASIR remained relatively stable, with a slight increase. Conversely, the global ASIR for GBDT demonstrated a consistent downward trend throughout the same period. From 1990 to 2021, the ASMR for GBDT remained relatively low both in China and worldwide, showing stable trends without significant changes. Similarly, the ASDR for GBDT in China and worldwide were also low but gradually declined annually (Figure 1).

### 3.3 Joinpoint regression analysis of the burden of GBDT in China and worldwide

**Figure 2:**
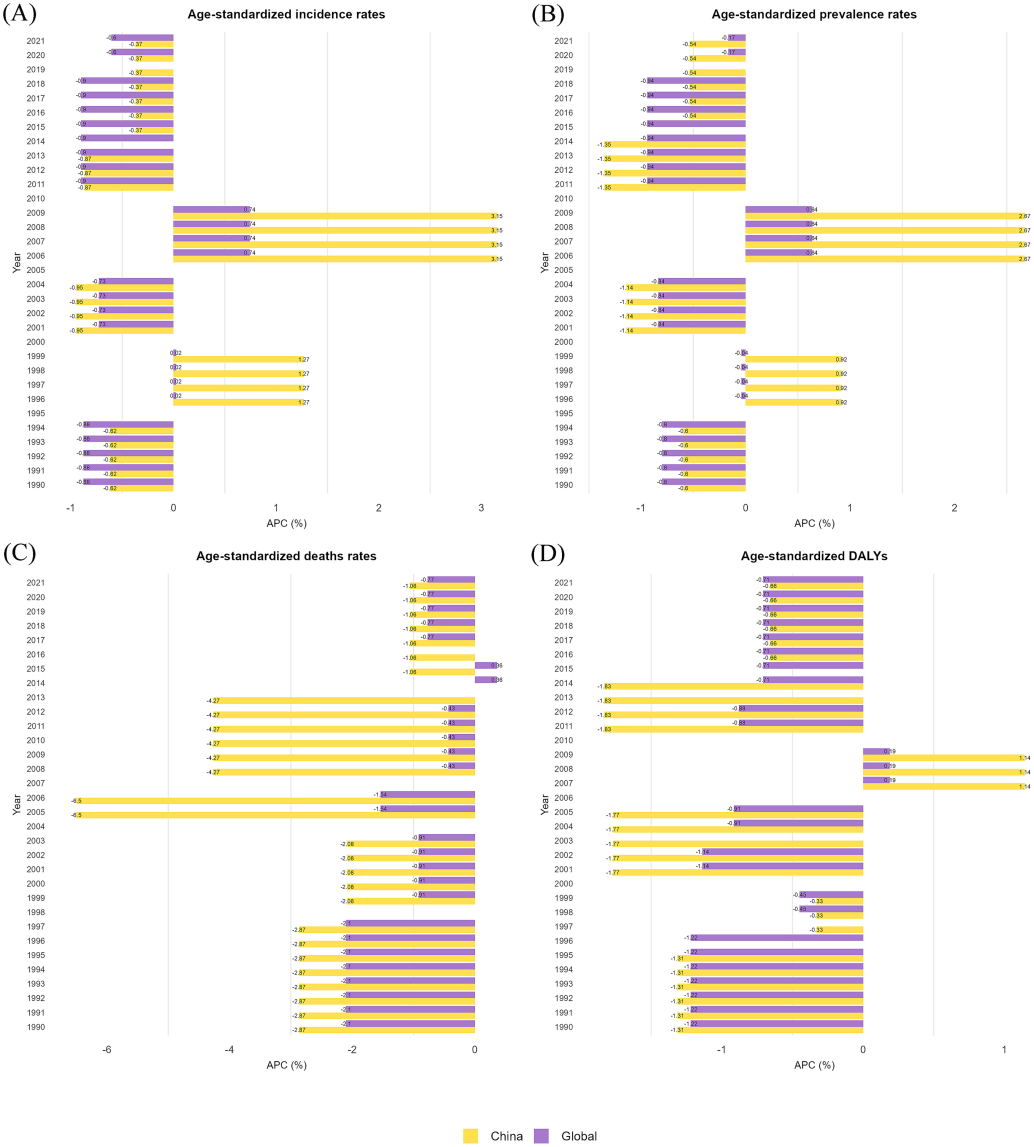
Annual percentage changes (APCs) in the ASIR, ASPR, ASMR, and ASDR for GBDT in China and globally from 1990–2021. **(A)** ASIR; **(B)** ASPR; **(C)** ASMR; **(D)** ASDR.

Figure 2 depicts a dual-sided graph generated from joinpoint regression analysis, showing the ASIR, ASPR, ASMR, and DALYs for GBDT in both China and globally from 1990 to 2021. The figure highlights the trends in the annual percentage change (APC). Both the ASIR for China and the global GBDT significantly declined from 1990 to 1995 (*p* < 0.05). However, from 1995 to 2000, China’s ASIR significantly increased (*p* < 0.05), whereas the global rate remained stable. From 2000 to 2005, both China and the global ASIR experienced significant declines (*p* < 0.05). In the following period, from 2005 to 2010, both rates rose significantly (*p* < 0.05), with China’s ASIR in 2010 exceeding the 1990 levels. After 2010, the ASIR experienced a steady decline (*p* < 0.05, Figure 2A).

The ASPR for China and the global GBDT displayed similar trends (Figure 2B). China’s ASPR demonstrated notable fluctuations, following a clear downward trajectory after 1995 and 2005 (*p* < 0.05). It also experienced significant increases after 2000 and 2010 (*p* < 0.05). Conversely, the global ASPR generally showed a downward trend, with a brief period of sharp increase occurring only between 2005 and 2010 (*p* < 0.05).

From 1990 to 2021, both China and the global GBDT showed a declining trend in the ASMR (Figure 2C). China’s ASMR decreased most rapidly between 2004 and 2007 (APC = −6.50, *p* < 0.05), followed by a gradual decline after 2014 (APC = −1.06, *p* < 0.05). In comparison, the global ASMR declined more steadily, with the steepest reduction occurring from 1990 to 1998 (APC = −2.10, *p* < 0.05), before stabilizing after 2007.

The ASDR for both China and the global GBDT exhibited an overall decline from 1990 to 2021 (Figure 2D). Only between 2006 and 2010 were there varying degrees of increase, which was particularly pronounced in China (*p* < 0.05). A review of the dual-sided graphs shows that significant changes in China’s GBDT-related ASIR, ASPR, ASMR, and ASDR correspond with similar directional trends in global metrics, indicating shifts between increasing and decreasing patterns. This connection highlights the link between the disease burden in China and global trends, particularly because China is the most populous country. Additional line graphs from the joinpoint regression analysis can be found in the Supplementary materials.

### 3.4 The burden of GBDT in different age groups in China in 1990 and 2021

**Figure 3:**
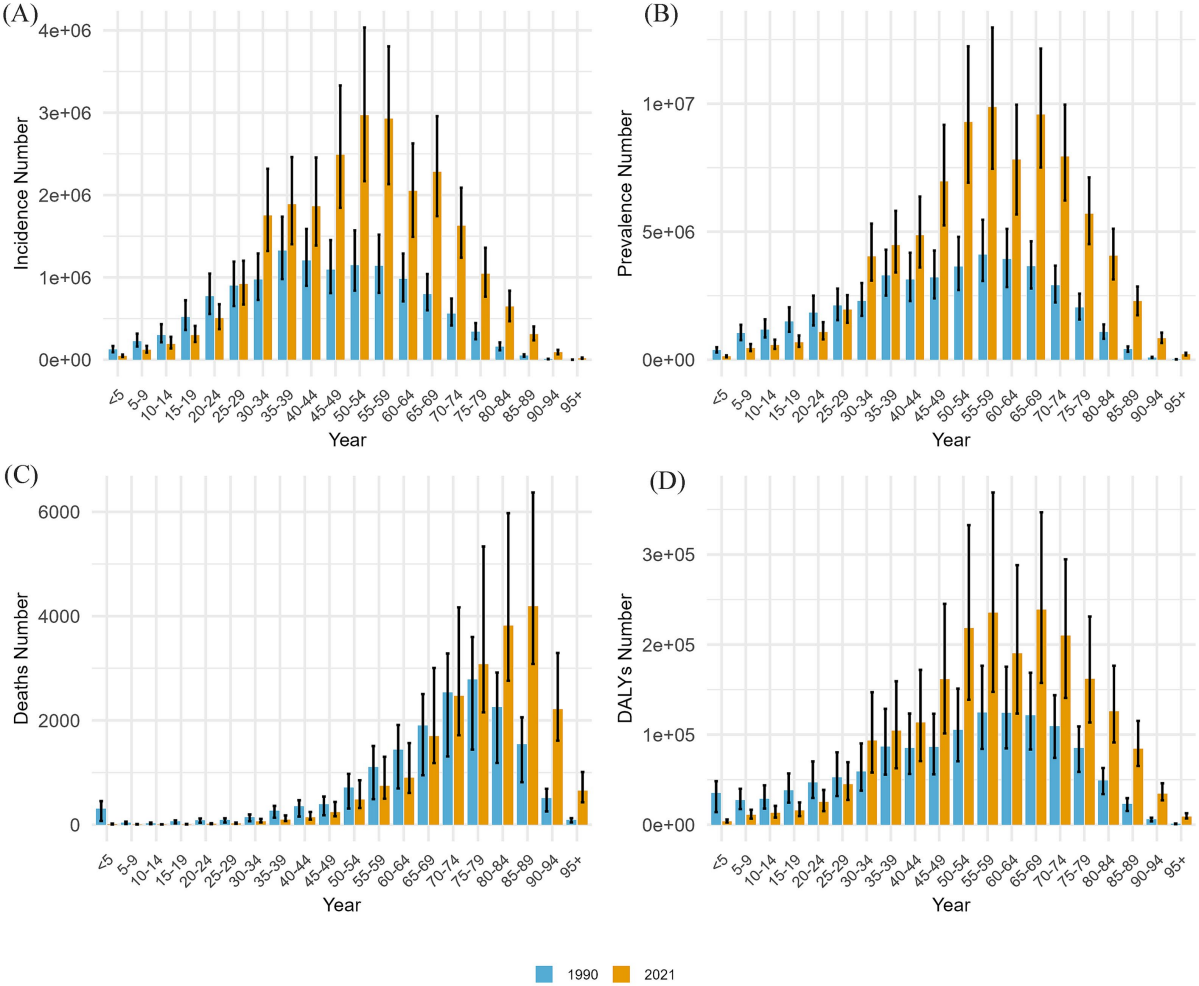
This figure compares the incidence, prevalence, number of deaths, and DALY counts for different age groups in China from 1990 to 2021. The following sections are included: **(A)** incidence cases; **(B)** prevalence cases; **(C)** death cases; and **(D)** counts of DALYs.

Figure 3 presents a comparison of the incidence, prevalence, mortality, and DALYs of GBDT across various age groups in China, covering the period from 1990 to 2021. This comparison also includes the crude rates for these measures, providing a clear depiction of the epidemiological trends and health burden of GBDT over the 31-year interval. GBDT impacts people of all age groups, with the incidence of cases increasing as individuals age from birth. In 1990, the 35–39 years age group reported the highest number of cases, whereas by 2021, this number shifted to the 50–54 years age group, after which the number of cases declined. The crude incidence rate for GBDT demonstrated a steady increase across age groups, rising from those under 5 years to 85–89 years in both analyzed years, with a decline observed after the age of 90. Notably, in 1990, the highest incidence rate was recorded in the 80–84 years age group, whereas by 2021, the age group with the highest incidence rate was delayed by 5 years. All 19 + age groups had higher incidence rate in 2021 than they did in 1990 (Figure 3A).

The crude prevalence rate for GBDT also displayed an increasing trend across all age groups in both 1990 and 2021, with the highest prevalence observed in the 55–59 age group (Figure 3B).

In terms of mortality, the age group with the most deaths transitioned from 75 to 79 years in 1990 to 85–89 years in 2021. For individuals aged 75 and older, GBDT-related deaths in 2021 exceeded those recorded in 1990. While the crude mortality rate increased with age, it is important to note that for those aged 65–69 years and older, the mortality rate was higher in 1990 than in 2021. Throughout both years, the 95 years and older age group consistently presented the highest mortality rate (Supplementary Figure S1C).

With respect to DALYs, in 1990, the highest number of DALYs was observed in the 55–59 age group, whereas in 2021, the peak shifted to the 65–69 age group (Figure 3D). The crude DALY rate increases progressively with age, accelerating significantly after age 85, with 1990 data generally exceeding 2021 data for all age groups. (Supplementary Figure S1D).

### 3.5 Gender disparities in the burden of GBDT across various age groups in China

**Figure 4:**
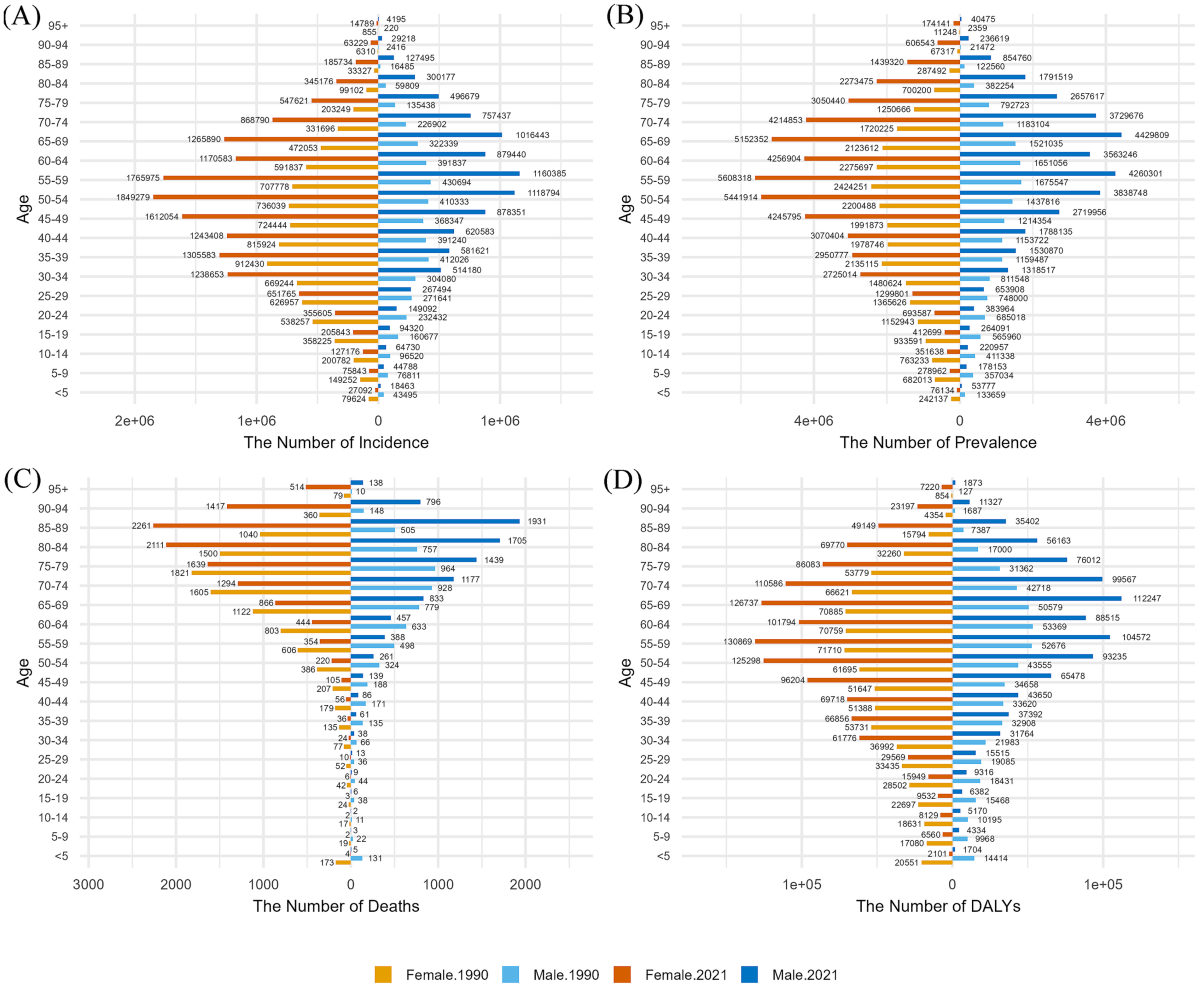
Comparison of the number of cases, prevalence, deaths, and DALYs of GBDT for males and females in different age groups in China in 1990 and 2021, grouped into five-year intervals. **(A)** Incidence **(B)** prevalence; **(C)** deaths; **(D)** DALYs.

The incidence data presented in Figure 4A reveal that GBDT occurred more in females than in males across all age groups in both 1990 and 2021. Additionally, for individuals older than 29 years, the incidence in 2021 exceeded that in 1990. In both 1990 and 2021, males had the greatest number of GBDT episodes in the 55–59 age group, whereas females had the greatest number of episodes in the 35–39 age group in 1990 and in the 50–54 age group in 2021.

The prevalence results shown in Figure 4B indicate that GBDT primarily affects middle-aged individuals. In 1990, the highest prevalence for both sexes was in the 55–59 years age group; however, in 2021, females maintained their peak prevalence in this group, whereas males peaked in the 65–69 years age group. Consistent with the incidence trends, females presented a greater prevalence of GBDT than males across all age groups. Additionally, the prevalence figures in 2021 were gradually higher than those in 1990 as age increased.

The mortality trends for GBDT are compared between sexes in Figure 4C. In 1990, mortality peaked in the 75–79 years age group for both males and females, subsequently decreasing with age. By 2021, the peak mortality rate had shifted to 85–89 years for both sexes. Notably, female mortality surpassed that of 1990 for individuals aged 79 and older, whereas the mortality of males increased starting at age 64. In 2021, males under 65 years of age experienced higher mortality rate than females did, except in the 10–14 years age group. Conversely, males over 65 years had lower mortality rate than females did—in contrast to 1990, when male mortality was consistently lower than female mortality in all age groups above 40 years. Before age 40, both experienced their own highs and lows in mortality rate.

The results for DALYs shown in Figure 4D exhibit a spindle shape, revealing that females had higher DALYs than males across all age categories. In 1990, women had the highest number of DALYs in the 55–59 age group, whereas the peak for men was delayed by 5 years to the 60–64 age group. By 2021, females maintained their highest DALYs in the 55–59 years age group, whereas males reached their peak in the 65–69 years age group. In addition, as shown in Figure 4, the incidence, prevalence, and DALYs for both men and women in the 60-65-year-old group in 2021 were lower than those for the adjacent age groups on either side, a trend not evident in 1990.

### 3.6 The disease burden of GBDT in China by sex from 1990 to 2021

**Figure 5:**
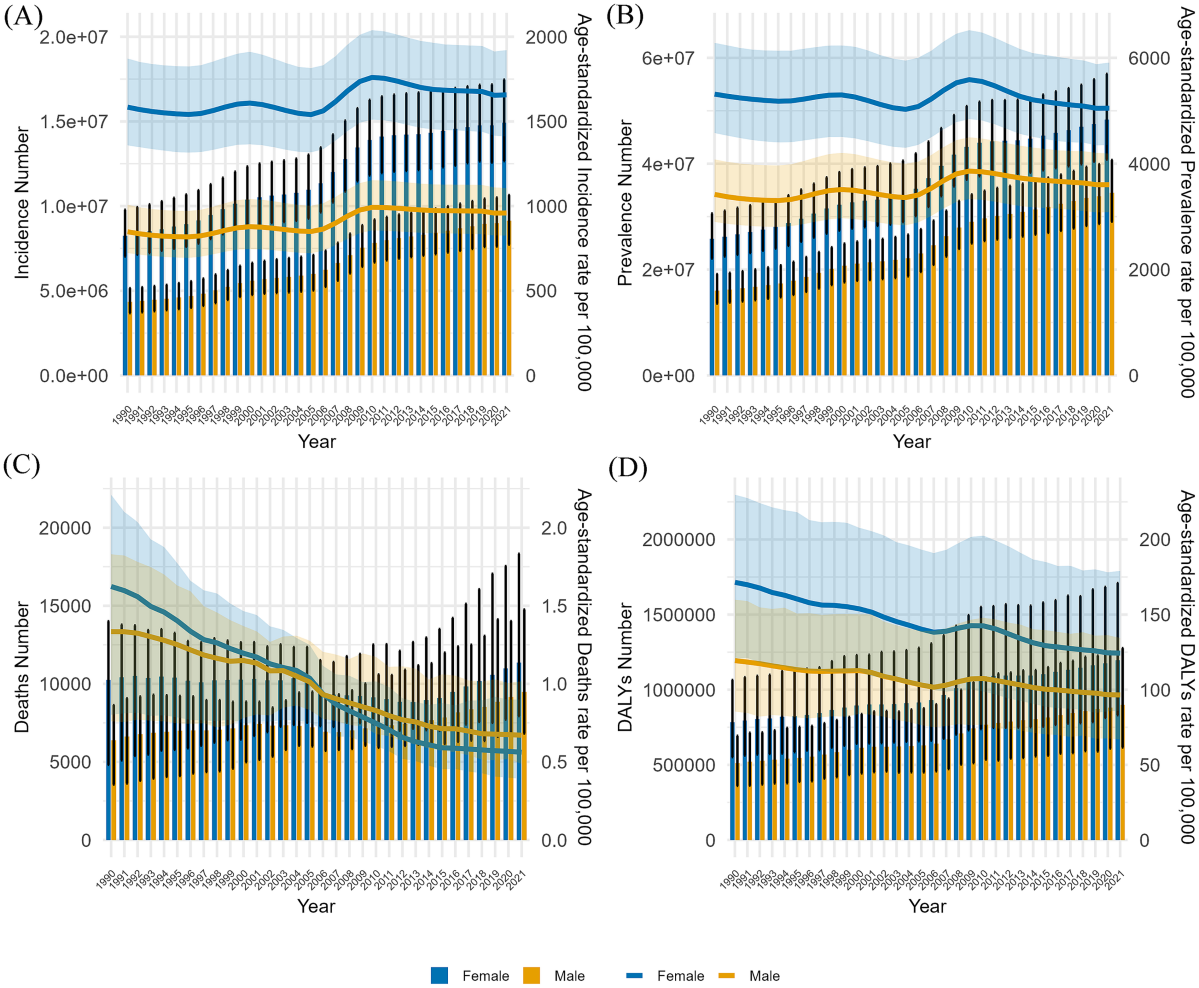
This report compares the number of cases in all age groups and the ASIR, APSR, ASMR, and ASDR for males and females in China from 1990–2021. The analysis includes **(A)** incident cases and ASIRs, **(B)** prevalent cases and ASRs, **(C)** mortality cases and ASMRs, and **(D)** the number of DALYs and ASDRs. Bar charts depict counts, while line graphs illustrate age-standardized rates.

Figure 5 presents the disease burden of GBDT among all age groups by sex in China from 1990–2021, including age-standardized rates. Figure 5A shows the ASIRs of GBDT for both genders, revealing similar trends characterized by fluctuations. From 1990 to 1995, there was a gradual decline, followed by a moderate increase from 1996 to 2000 and another decrease from 2000 to 2005. Notably, a significant increase in the ASIRs occurred between 2006 and 2010, followed by a slow decline over the past decade. Throughout this period, the ASIRs for females consistently exceeded that for males, with overall new cases rising annually and female cases outpacing male cases. The ASPR for both sexes also fluctuated from 1990–2021, reflecting a pattern similar to that of the ASIR. Annual case numbers increased for both genders, with female ASPRs and prevalent cases consistently higher than those of males (Figure 5B).

In contrast, the ASMRs for both males and females showed a distinct downward trend between 1990 and 2021, with a more rapid decline observed in females. The gap in the ASMR between genders narrowed from 1990 to 2006. After this period, the female ASMR shifted from being higher than the male ASMR to being lower. Throughout this timeframe, the overall number of deaths for both genders exhibited only minor fluctuations without a significant trend (Figure 5C). While there was a slight increase in the ASDRs for females between 2006 and 2009 and modest increases for males from 1996 to 2000 and again from 2006 to 2010, the overall trend decreased. Both sexes experienced a gradual annual increase in DALYs.

### 3.7 Risk factors for GBDT

On the basis of the GBD 2021 database, the influence of behavioral and metabolic factors on the incidence of GBDT increases with age, with metabolic factors exerting a significantly greater impact than environmental factors. Between 1990 and 2021, high BMI consistently emerged as the leading risk factor among metabolic influences, with its effect on GBDT substantially increasing from 19.18 DALYs per 100,000 in 1990 to 48.6 DALYs per 100,000 in 2021, an increase of 153.46%.

#### 3.7.1 Deaths and DALYs due to HBMI

**Figure 6:**
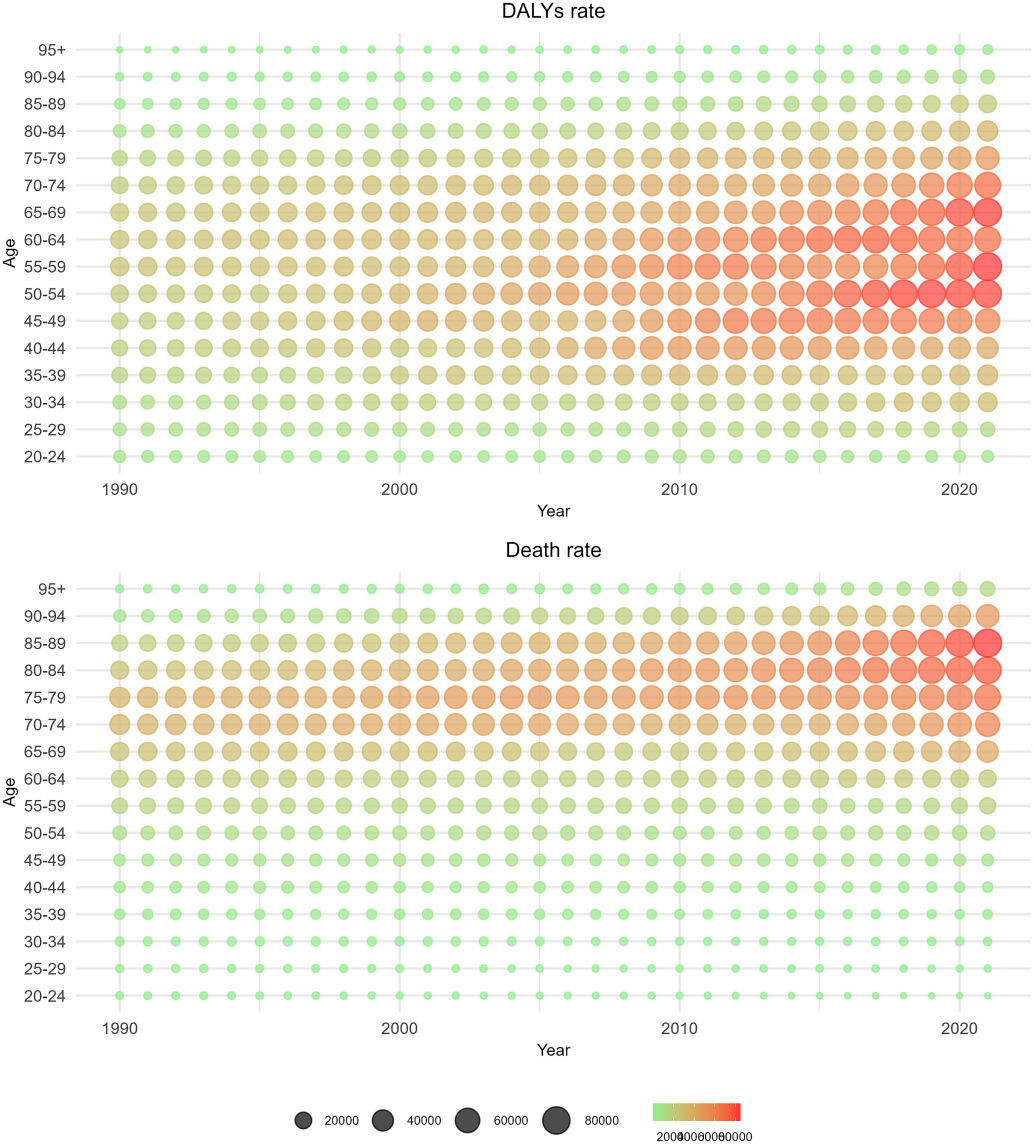
Distribution of the number of deaths and the number of DALYs from GBDT attributed to HBMI by age group from 1990 to 2021.

Figure 6 illustrates a gradual increase in DALYs from GBDT associated with HBMI over the years. This trend is particularly pronounced in individuals aged 50–69 years, where the impact of HBMI is most significant. In contrast, deaths related to GBDT and HBMI are predominantly observed in the older adult population aged 75 years and older over the past 5 years, with comparatively few fatalities reported in those aged 95 years and older.

**Figure 7:**
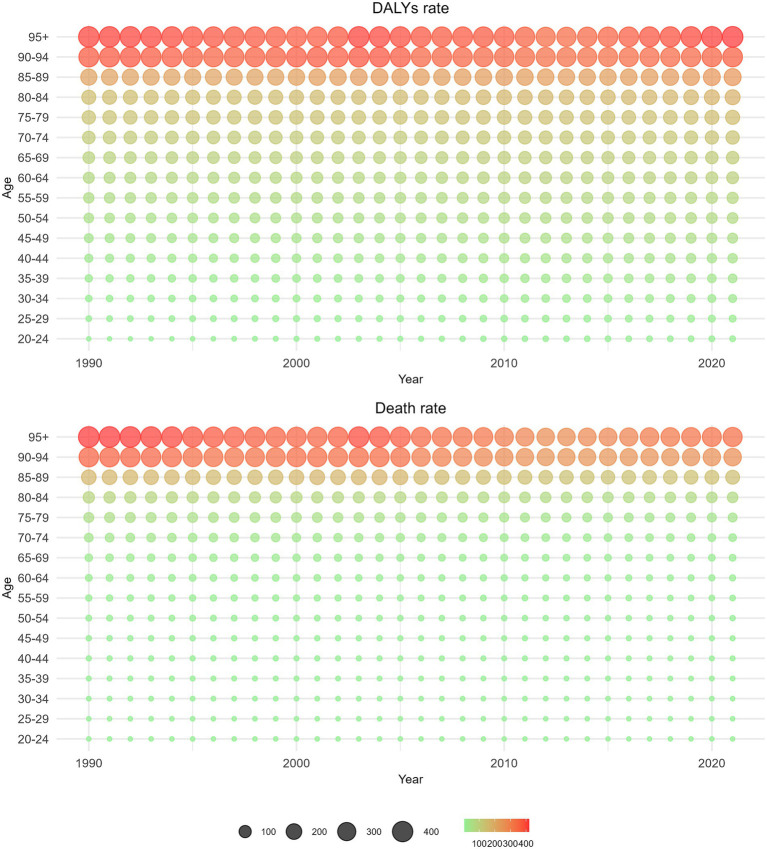
Distributions of death rate and DALYs rate from GBDT attributed to HBMI by age group from 1990 to 2021.

Figure 7 reveals that as age increases, the rate of DALYs from GBDT linked to HBMI also increases steadily, especially among individuals aged 75 years and above. The highest increase in DALYs due to HBMI occurred in the 95-year-old and older age groups. Additionally, the mortality rate from GBDT associated with HBMI tends to increase with age, significantly increasing in those aged 85 years and above and reaching its peak in those 95 years and older. Analysis on the basis of balloon size and density revealed slight fluctuations in the mortality rate over time, accompanied by a gradual increase over the past 5 years.

#### 3.7.2 Projections of age-standardized deaths and burden of disease in GBDT due to HBMI over the next 15 years

**Figure 8:**
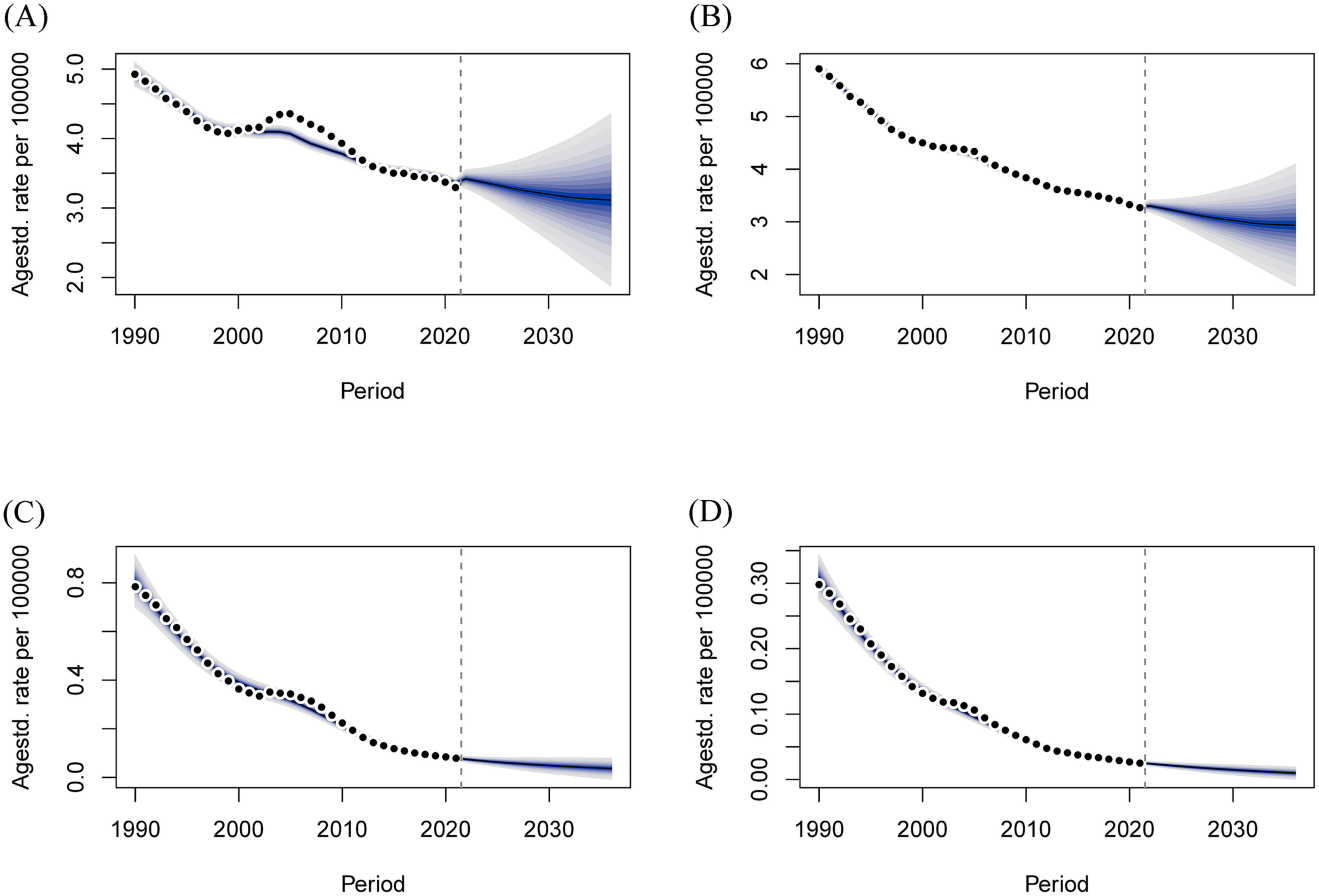
Projections of age-standardized deaths and deaths over the next 15 years for GBDT attributable to HBMI in men and women. **(A)** Number of male deaths, **(B)** Number of female deaths, **(C)** Male death rate, and **(D)** Female death rate.

**Figure 9:**
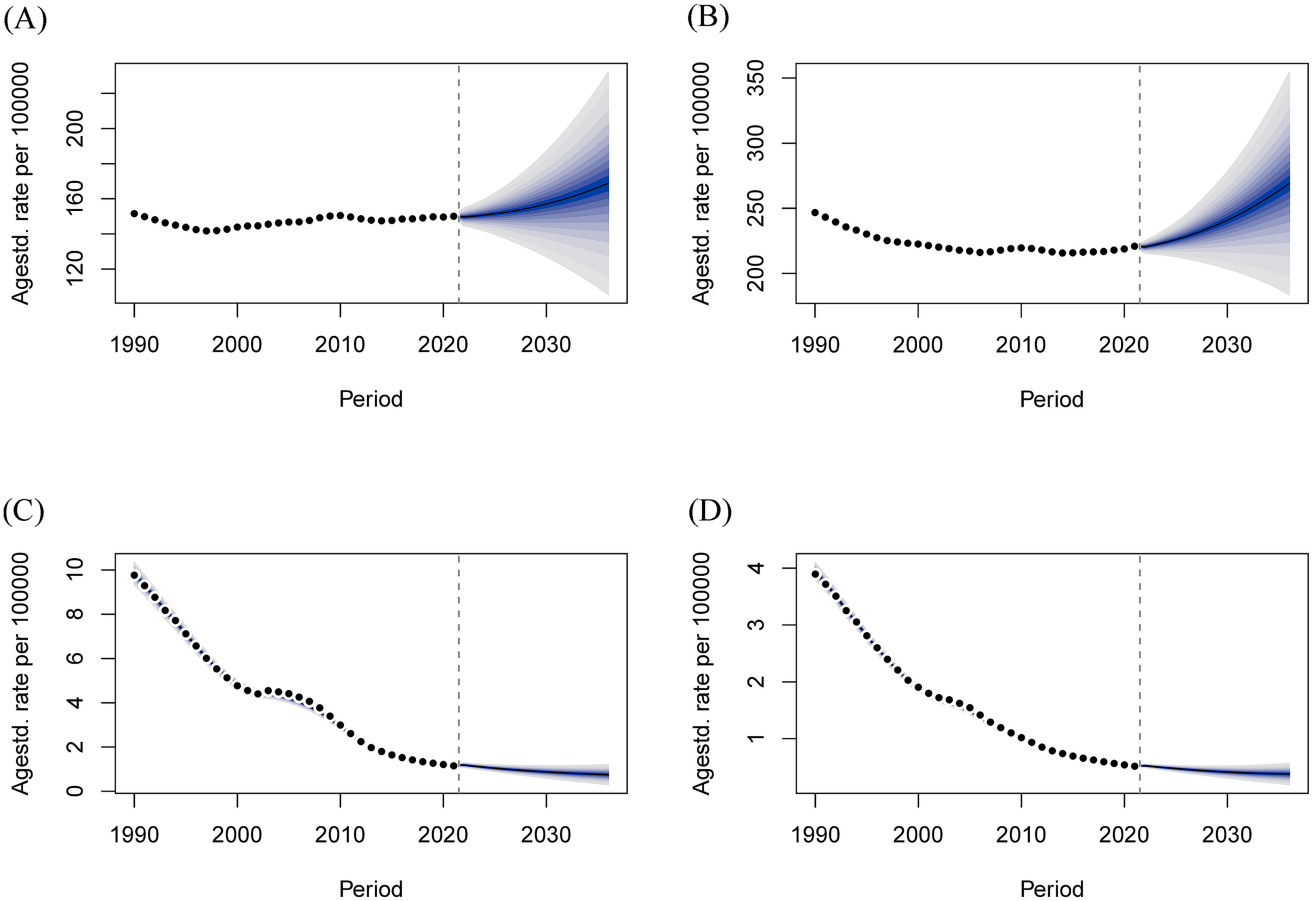
Projections of age-standardized rate of DALYs and number of DALYs over the next 15 years for males and females with GBDT attributable to HBMI. **(A)** Male DALYs number, **(B)** Female DALYs number, **(C)** Male DALYs rate, **(D)** Female DALYs rate.

The key assumptions underlying the 15-year projection are as follows: there will be no breakthroughs in medical technologies (such as the further dissemination and universal adoption of screening), no public health interventions (such as new screening guidelines), and no alterations in the living environment. The BAPC model revealed that over the next 15 years, the number of deaths from GBDT due to HMBI in both males and females, although elevated in the short term, showed an overall decreasing trend, and the decrease was greater in females than in males. Over the next 15 years, mortality rate for both males and females showed a gentle downward trend as well (Figure 8). In terms of DALYs, the number of DALYs due to HBMI-induced GBDT trended upward over the next 15 years in both men and women, whereas DALYs rate slowly declined (Figure 9).

Furthermore, our analysis compared the distribution of GBDT-related mortality and DALYs attributable to HBMI by sex and age group in China. The results indicated that middle-aged and older adult populations exhibited higher mortality rates and greater disease burdens (Supplementary Figures S6–S9).

## 4 Discussion

### 4.1 Key findings

This study offers a comprehensive assessment of the incidence, prevalence, deaths, and DALYs associated with GBDT in China and globally over the past 31 years, utilizing data from the GBD 2021 database.

The findings revealed that the ASPR and ASIR of GBDT in China exhibited fluctuating trends from 1990 to 2021. Similar fluctuations were observed globally during the same period; however, the magnitude of these changes was significantly smaller, with an overall decreasing trend. The ASMR remained low and stable, while the ASDR also remained low, gradually decreasing over time.

GBDT incidence, prevalence, mortality, and DALYs correlate with patient age, increasing notably in the older adult population, particularly mortality. With respect to sex, females are more frequently affected by GBDT, resulting in a greater overall burden. However, ASMRs was higher in females than in males prior to 2006, whereas the trend reversed after 2006. This is in contrast to the global trend where ASMRs have consistently been higher in females than in males (16). The disease burden from GBDT linked to HBMI is particularly pronounced in the older population. The BAPC model predicted a decline in the number of deaths and DALYs rate due to GBDT attributable to HBMI over the next 15 years, as well as an increase in the number of DALYs and female mortality.

Additionally, joinpoint regression analysis revealed significant similarities in the ASIR and ASPR trends between China and global figures, particularly at critical time points, suggesting a strong connection between China, the most populous country, and global trends in GBDT.

Notably, since the initiation of China’s national basic public health service program in 2009 (26), which focuses on health education and management, the increase in GBDT prevalence has slowed. This deceleration is attributed to improved public awareness of the disease. Additionally, when adjusting for population growth and aging via age-standardized rates, the prevalence of GBDT has demonstrated a downward trend since 2010.

### 4.2 Epidemiologic features of GBDT in global and China

GBDT exhibits significant geographical heterogeneity globally, with its incidence and risk factors closely associated with regional economic levels, dietary habits, and public health policies. Global gallstone prevalence reaches 6.1%, with South America reaching 11.2% - significantly higher among females than males (27). China’s standardized prevalence is 5.13%, comprising gallbladder stones (76.3%), intrahepatic bile duct stones (24.3%), and extrahepatic bile duct stones (0.2%) (28).

East Asia is a high-prevalence region for primary hepatolithiasis (oriental cholangitis), where the prevalence in Taiwan, China once reached 20%, while secondary stones (e.g., complications from primary sclerosing cholangitis or biliary surgery) dominate in Western countries with incidence generally below 2% (29). Research from Beijing Children’s Hospital in China showed that the hospitalization incidence rate for pediatric primary hepatolithiasis was only 1.7 per 10,000, indicating its rarity among children (30). Unlike cholesterol gallstones in cholecystolithiasis, primary hepatolithiasis primarily consists of brown pigment gallstones composed of calcium bilirubinate, where bile metabolism appears to play a central role in intrahepatic stone development. Parasitic infections such as roundworms represent a unique etiological factor (31).

In contrast, another autoimmune disease, primary sclerosing cholangitis, shows higher prevalence in Northern Europe and North America, with incidence and prevalence rates increasing over time. Finland’s prevalence rose from 3.85 to 31.7 per 100,000 between 1990 and 2015, while China’s overall prevalence remains lower (2.36/100,000) but has significantly increased post-2010, potentially related to improved diagnostic capabilities and economic development, though no BMI or weight impact was observed (32, 33). However, a two-sample Mendelian randomization study in China demonstrated that BMI serves as a causative factor for primary biliary cholangitis, with its prevalence rising from 2.16 per 100,000 during 1991–2000 to 8.99 per 100,000 in 2011–2020 (34, 35).

### 4.3 Drivers of temporal trends

Between 2005 and 2010, advancements in medical technology and healthcare optimization led to increased detection of previously undiagnosed biliary tract and gallbladder diseases. During this period, a surge in disease reporting occurred due to enhanced diagnostic sensitivity. Over the years, the number of healthcare facilities in China has dramatically increased, growing from 2,803 in 1950 to 997,433 by 2018, reflecting an expansion of more than 110 times (34). Moreover, China has seen substantial improvements in the HAQ (Healthcare Access and Quality) Index, with a notable increase of 35.3 points from 1990 to 2016, marking the fastest improvement among 195 countries and regions during this time. In particular, from 2000 to 2016, the HAQ index rose by 24.6 point (36). Concurrently, health literacy among Chinese citizens has progressively improved; by 2023, the health literacy rate reached 29.70%. This metric encompasses an understanding of basic health concepts, healthy lifestyles and behaviors, and fundamental health skills. Increased health literacy has enhanced disease prevention, diagnosis, and treatment awareness, contributing to a decline in GBDT incidence after an initial increase from 2005 to 2010 (37, 38). The increase in hospital admissions, from 0.22 billion in 1980 to 0.2 billion in 2018, also illustrates these trends (37). Despite the increasing annual prevalence rates, the ASMR has remained relatively low, which can be attributed to China’s rapid advancements in healthcare infrastructure development and improvements in medical standards.

Recent statistics from China’s National Bureau of Statistics indicate a notable demographic change, revealing that as of January 17, 2022, 267.36 million individuals aged 60 and older constitute 18.90% of the total population. Among these, 200.56 million are 65 years or older, representing 14.2% of the national populace. This demographic detail confirms that China has deeply entered an advanced stage of population aging, defined by more than 14% of the population being 65 years or older (39). GBDT clearly tends to increase in prevalence, mortality, and disease burden with age, which is consistent with findings from both 1990 and 2021 (7, 40). Our analysis has unraveled crucial disparities in the epidemiology of GBDT between the global paradigm and that of China. On a global scale, the ASIR has shown a decline, concurrently with an upward trend in the number of incident cases (Table 1). This phenomenon underscores the preeminent role played by population growth and aging in propelling the expansion of the disease burden. Notably, this occurs despite a marginal reduction in the age-adjusted disease risk. This pattern is consistent with the far-reaching influence of demographic transition on non-communicable diseases across the globe. In marked contrast, during the period from 1990 to 2021, China has exhibited a synchronous increase in both ASIR and the absolute incidence. This dual growth suggests that changes in population structure alone are insufficient to explain the escalating GBDT burden in China. Instead, it mirrors a tangible elevation in disease risk at the population level and enhanced screening efforts. This divergence positions China as a distinctive setting for epidemiological research. Specifically, the influence intensity of specific risk factors (notably the surge in obesity rates and the transformation of dietary patterns) on the pathogenesis of GBDT surpasses the levels commonly observed globally. However, our findings should be interpreted with caution in individuals aged 95 years or older due to potential underdiagnosis caused by limited mobility or the presence of comorbidities. With increasing age, hepatic cholesterol secretion increases, while bile acid synthesis decreases. This can lead to reduced cholesterol utilization in the liver and an increase in cholesterol saturation in bile, thereby raising the risk of cholesterol stone development in the older adult population (41). Older patients are particularly susceptible to severe outcomes due to increased comorbidities, rapid disease progression, and lower tolerance for surgical interventions. Surgical risks are compounded by higher rates of postoperative complications and mortality, primarily due to diminished immune responses and stress tolerance in older adult individuals.

The primary lethal outcomes of GBDT are caused by obstructions in the biliary system, leading to sepsis and uncontrollable infections (42). Medical advancements over the last three decades have significantly improved the management of GBDT. Since the first independent laparoscopic cholecystectomy in China in 1991, this minimally invasive technique has expanded widely, becoming the norm in many clinical settings and constituting more than 80% of such surgeries in some hospitals (37). Additionally, innovations such as 3D visualization, 3D printing, mixed reality displays, and indocyanine green fluorescence imaging have revolutionized hepatobiliary surgeries, enhancing precision, minimizing intraoperative blood loss, and reducing overall physical trauma (39). These advancements in diagnosis and treatment have contributed to a steady decrease in GBDT DALYs rates in China, with projections from the BAPC model indicating that this decline will continue over the next 15 years.

The global burden of disease due to obesity is increasing yearly (43), and the BAPC projections show the impact of obesity on the burden of disease in GBDT, with number of DALYs trending upward over the next 15 years. Obesity serves as a core risk factor for gallstones, with individuals having a BMI ≥ 30 kg/m^2^ exhibiting a 3.02-fold higher risk compared to those of normal weight. Visceral fat accumulation (e.g., elevated waist-to-hip ratio) further exacerbates this risk (44). Since 2014, China has become the country with the largest obese population globally (45). From 1961 to 2009, global per capita consumption of meat, refined sugar, and total calories increased significantly with rising incomes, with China experiencing particularly rapid growth—surpassing the global average and continuing to expand post-2009. This high-sugar, high-fat, high-meat dietary pattern has promoted obesity and metabolic disorders, explaining why China’s GBDT prevalence has risen markedly faster than in other upper-middle-income regions (16, 46).

While the obesity rate among Chinese men exceeds that among women (11), the incidence and prevalence of GBDT are higher in women than in men, which aligns with findings from previous studies (7, 47, 48). Globally, the prevalence of cholelithiasis is higher in women than in men across different countries and regions (49), which is consistent with our global-scale study. Estrogen and progesterone receptors are expressed in gallbladder tissue (50). Obese women exhibit reduced levels of sex hormone-binding globulin, lower bioavailability of sex hormones, and elevated concentrations of both estrogens and androgen (51). Estrogen can contribute to the formation of cholesterol stones through several pathways (52).

The sales of ultra-processed foods and sugary beverages in China have surged notably, with the number of fast-food chains increasing sixfold between 2004 and 2018, while domestic demand for empty calories like refined sugar and processed fats has progressively increased. This convergence of factors further elevates obesity rates among the population and indirectly drives the development of GBDT (46, 53). Although China initiated the China Health and Nutrition Survey in 1989 for long-term tracking and research, efforts to control healthy diets have remained limited to promotional guidance and health education, advocating balanced diets and reduced sugar intake without implementing targeted policies on sugary drinks or other foods. In 2010, China’s Ministry of Health released the Nutrition Improvement Work Management Measures, clarifying responsibilities for nutrition monitoring, education, and intervention. Simultaneously, the Ministry of Education introduced the “One Hour of Daily Campus Physical Activity” policy. These measures contributed to obesity reduction and may indirectly explain the declining trends in GBDT prevalence and incidence observed after 2010 in this study (54).

### 4.4 Implications for health policies

Considering the aforementioned findings, China’s disease burden of GBDT is influenced by multiple factors, including population aging, the obesity epidemic, and gender heterogeneity. In recent years, several measures have been implemented to address GBDT. In 2019, China established a standardized database for hepatobiliary diseases, which has significantly advanced disease prevention, early screening, and standardized treatment in this field (55). In 2021, Wei et al. proposed a method using deep learning on ocular images to screen and identify hepatobiliary diseases, offering an innovative approach for GBDT detection (56). In 2024, the Shanghai Municipal Health Commission of China recommended measures for gallstone prevention, including weight control, increased physical activity, and reduced saturated fatty acid intake (57). Also in 2024, China launched a three-year “National Weight Management Year” campaign aimed at improving public awareness and skills related to weight management (58).

While advancements in medical technology and public health policies have partially mitigated the disease burden, targeted interventions remain essential to address future challenges. To this end, the following measures are recommended: (1) Integrate gallbladder ultrasound into annual free health checkups, extending coverage to underserved rural areas. Prioritize embedding advanced medical technologies in primary care facilities, with a focus on obese and female patients. (2) Enhance public education on rational dietary habits and systematic weight management, while providing incentives (e.g., grants or recognition) for social media platforms to promote relevant content through videos or articles. (3) Expand laparoscopic training programs to facilitate the widespread adoption of laparoscopic cholecystectomy. (4) Implement rational taxes on high-sugar and high-fat foods to reduce consumption and indirectly curb intake of such products, thereby supporting weight control.

### 4.5 Limitations and future perspectives

This study has several limitations that impact the reliability of the GBD estimates. The accuracy of these estimates is contingent upon the quality and comprehensiveness of the data, which requires precise disease diagnosis and thorough evaluation of long-term environmental risks. Notably, although GBDT involve multifactorial etiology (including dietary patterns and region-specific parasites like *Clonorchis sinensis*), these factors are not incorporated in the GBD 2021 risk assessment framework. Economically disadvantaged areas often face challenges such as limited healthcare access and economic disparities, leading to frequent misdiagnosis or underdiagnosis of diseases and, consequently, an underestimation of the true disease burden. Moreover, issues with diagnostic accuracy and coding may compromise the integrity of the data. Variability in diagnostic techniques and data collection methods across different times and regions can also introduce biases, underscoring the need for standardized diagnostic criteria to increase data precision.

While this study provides insights into the global and Chinese perspectives on GBDT, highlighting the importance of China’s vast population, it fails to account for several unique factors that could influence disease burden on a global scale. These factors include socioeconomic status, geographic and environmental conditions, genetics, ethnicity, and medical resource availability. Additionally, the study does not address the time lag in updating the GBD database, which may limit the applicability of these global data as a reference for assessing the disease burden in specific countries or regions. Future studies should therefore utilize national surveillance data to capture region-specific risk factors (e.g., parasite prevalence). A more targeted and comprehensive analysis using actual data from individual countries or regions is essential for a clearer understanding of the disease burden.

## Data Availability

Publicly available datasets were analyzed in this study. This data can be found here: http://ghdx.healthdata.org/gbd-results-tool.

## Footnotes

^1^ http://ghdx.healthdata.org/gbd-results-tool

## References

[1] Peery AF Crockett SD Murphy CC Dellon ES Lund JL Williams JL. Burden and cost of gastrointestinal, liver, and pancreatic diseases in the United States: update 2021. Gastroenterology. (2022) 162:621–44. doi: 10.1053/j.gastro.2021.10.017 34678215 PMC10756322

[2] Sandler RS Everhart JE Donowitz M Adams E Cronin K Goodman C. The burden of selected digestive diseases in the United States. Gastroenterology. (2002) 122:1500–11. doi: 10.1053/gast.2002.32978, PMID: 11984534

[3] Wilkins T Agabin E Varghese J Talukder A Franks AM Diehl DL. Gallbladder dysfunction: cholecystitis, choledocholithiasis, cholangitis, and biliary dyskinesia. Prim Care. (2017) 44:575–97. doi: 10.1016/j.pop.2017.07.002 29132521

[4] Unalp-Arida A Ruhl CE. Increasing gallstone disease prevalence and associations with gallbladder and biliary tract mortality in the US. Hepatology. (2023) 77:1882–95. doi: 10.1097/HEP.0000000000000264, PMID: 36631004

[5] Fujita N Yasuda I Endo I Isayama H Iwashita T Ueki T. Evidence-based clinical practice guidelines for cholelithiasis 2021. J Gastroenterol. (2023) 58:801–33. doi: 10.1007/s00535-023-02014-6, PMID: 37452855 PMC10423145

[6] Luo X Yang W Joshi AD Wu K Simon TG Yuan C. Gallstones and risk of cancers of the liver, biliary tract, and pancreas: a prospective study within two U.S. cohorts. Br J Cancer. (2022) 127:1069–75. doi: 10.1038/s41416-022-01877-5, PMID: 35715632 PMC9470543

[7] Su Z Gong Y Liang Z. Prevalence of gallstone in mainland China: a meta-analysis of cross-sectional studies. Clin Res Hepatol Gastroenterol. (2020) 44:e69–71. doi: 10.1016/j.clinre.2020.04.015, PMID: 32446673

[8] Gallaher JR Charles A. Acute cholecystitis: a review. JAMA. (2022) 327:965–75. doi: 10.1001/jama.2022.2350 35258527

[9] Lee KJ Park SW Park DH Cha HW Choi A Koh DH. Gallbladder perforation in acute acalculous vs. calculous cholecystitis: a retrospective comparative cohort study with 10-year single-center experience. Int J Surg. (2024) 110:1383–91. doi: 10.1097/JS9.0000000000000994, PMID: 38079596 PMC10942242

[10] Kelly T Yang W Chen CS Reynolds K He J. Global burden of obesity in 2005 and projections to 2030. Int J Obes. (2008) 32:1431–7. doi: 10.1038/ijo.2008.102, PMID: 18607383

[11] Bai R Wu W Dong W Liu J Yang L Lyu J. Forecasting the populations of overweight and obese Chinese adults. Diabetes Metab Syndr Obes. (2020) 13:4849–57. doi: 10.2147/DMSO.S274110, PMID: 33324082 PMC7733397

[12] Mahase E. Global cost of overweight and obesity will hit $4.32tn a year by 2035, report warns. BMJ. (2023) 380:523. doi: 10.1136/bmj.p523, PMID: 36868577

[13] Liu J Wang H Wang Z Han W Hong L. The global, regional, and national uterine cancer burden attributable to high BMI from 1990 to 2019: a systematic analysis of the global burden of disease study 2019. J Clin Med. (2023) 12:1874. doi: 10.3390/jcm12051874, PMID: 36902661 PMC10003834

[14] Wang L Zhou B Zhao Z Yang L Zhang M Jiang Y. Body-mass index and obesity in urban and rural China: findings from consecutive nationally representative surveys during 2004-18. Lancet. (2021) 398:53–63. doi: 10.1016/S0140-6736(21)00798-4, PMID: 34217401 PMC7617101

[15] Kim HS Cho SK Kim CS Park JS. Big data and analysis of risk factors for gallbladder disease in the young generation of Korea. PLoS One. (2019) 14:e0211480. doi: 10.1371/journal.pone.0211480, PMID: 30794560 PMC6386282

[16] Li J Jin X Ren J Li R Du L Gao Y. Global burden of gallbladder and biliary tract diseases: a systematic analysis for the global burden of disease study 2019. J Gastroenterol Hepatol. (2022) 37:1389–99. doi: 10.1111/jgh.15859, PMID: 35430757

[17] Li ZZ Guan LJ Ouyang R Chen ZX Ouyang GQ Jiang HX. Global, regional, and national burden of gallbladder and biliary tract diseases from 1990 to 2019. World J Gastrointest Surg. (2023) 15:2564–78. doi: 10.4240/wjgs.v15.i11.2564 38111771 PMC10725539

[18] GBD 2021 Diseases and Injuries Collaborators. Global incidence, prevalence, years lived with disability (YLDs), disability-adjusted life-years (DALYs), and healthy life expectancy (HALE) for 371 diseases and injuries in 204 countries and territories and 811 subnational locations, 1990-2021: a systematic analysis for the global burden of disease study 2021. Lancet. (2024) 403:2133–61. doi: 10.1016/S0140-6736(24)00757-8, PMID: 38642570 PMC11122111

[19] GBD 2021 Risk Factors Collaborators. Global burden and strength of evidence for 88 risk factors in 204 countries and 811 subnational locations, 1990-2021: a systematic analysis for the global burden of disease study 2021. Lancet. (2024) 403:2162–203. doi: 10.1016/S0140-6736(24)00933-4, PMID: 38762324 PMC11120204

[20] GBD 2019 Diseases and Injuries Collaborators. Global burden of 369 diseases and injuries in 204 countries and territories, 1990-2019: a systematic analysis for the global burden of disease study 2019. Lancet. (2020) 396:1204–22. doi: 10.1016/S0140-6736(20)30925-9, PMID: 33069326 PMC7567026

[21] Hankey BF Ries LA Kosary CL Feuer EJ Merrill RM Clegg LX. Partitioning linear trends in age-adjusted rates. Cancer Causes Control. (2000) 11:31–5. doi: 10.1023/a:1008953201688, PMID: 10680727

[22] Mo ZY Qin ZZ Ye JJ Hu XX Wang R Zhao YY. The long-term spatio-temporal trends in burden and attributable risk factors of major depressive disorder at global, regional and national levels during 1990-2019: a systematic analysis for GBD 2019. Epidemiol Psychiatr Sci. (2024) 33:e28. doi: 10.1017/S2045796024000295, PMID: 38764153 PMC11362682

[23] Kim HJ Fay MP Feuer EJ Midthune DN. Permutation tests for joinpoint regression with applications to cancer rates. Stat Med. (2000) 19:335–51. doi: 10.1002/(sici)1097-0258(20000215)19:3<335::aid-sim336>3.0.co;2-z, PMID: 10649300

[24] Riebler A Held L. Projecting the future burden of cancer: Bayesian age-period-cohort analysis with integrated nested Laplace approximations. Biom J. (2017) 59:531–49. doi: 10.1002/bimj.201500263, PMID: 28139001

[25] Heng RS Chen WQ. Introduction to the age-period-cohort forecast model based on Bayesian methods [基于贝叶斯方法的年龄-时期-队列预测模型的介绍]. Chin J Prev Med. (2012) 46:648–50. doi: 10.3760/cma.j.issn.0253-9624.2012.07.016

[26] Li X Lu J Hu S Cheng KK De Maeseneer J Meng Q. The primary health-care system in China. Lancet. (2017) 390:2584–94. doi: 10.1016/S0140-6736(17)33109-4, PMID: 29231837

[27] Wang X Yu W Jiang G Li H Li S Xie L. Global epidemiology of gallstones in the 21st century: a systematic review and meta-analysis. Clin Gastroenterol Hepatol. (2024) 22:1586–95. doi: 10.1016/j.cgh.2024.01.051, PMID: 38382725

[28] Song Y Ma Y Xie FC Jin C Yang XB Yang X. Age, gender, geographic and clinical differences for gallstones in China: a nationwide study. Ann Transl Med. (2022) 10:735. doi: 10.21037/atm-21-6186, PMID: 35957733 PMC9358507

[29] Motta RV Saffioti F Mavroeidis VK. Hepatolithiasis: epidemiology, presentation, classification and management of a complex disease. World J Gastroenterol. (2024) 30:1836–50. doi: 10.3748/wjg.v30.i13.1836, PMID: 38659478 PMC11036492

[30] Yan J Zhang Z Guo J Lv C Chen Y. Clinical characteristics and prognosis of primary hepatolithiasis in hospitalized children. Eur J Pediatr. (2023) 182:3195–202. doi: 10.1007/s00431-023-05003-2, PMID: 37129614

[31] Lorio E Patel P Rosenkranz L Patel S Sayana H. Management of hepatolithiasis: review of the literature. Curr Gastroenterol Rep. (2020) 22:30–9. doi: 10.1007/s11894-020-00765-3, PMID: 32383039

[32] Trivedi PJ Bowlus CL Yimam KK Razavi H Estes C. Epidemiology, natural history, and outcomes of primary sclerosing cholangitis: a systematic review of population-based studies. Clin Gastroenterol Hepatol. (2022) 20:e4:1687–700. doi: 10.1016/j.cgh.2021.08.039 34474162

[33] Xu X Meng T Shi L Duan W Niu J Ding H. Prevalence and clinical profiles of primary sclerosing cholangitis in China: data from electronic medical records and systematic literature retrieval. J Autoimmun. (2024) 147:103264. doi: 10.1016/j.jaut.2024.103264, PMID: 38843578

[34] Trivedi PJ Hirschfield GM. Recent advances in clinical practice: epidemiology of autoimmune liver diseases. Gut. (2021) 70:1989–2003. doi: 10.1136/gutjnl-2020-322362, PMID: 34266966

[35] Xu H Wu Z Feng F Li Y Zhang S. Low vitamin D concentrations and BMI are causal factors for primary biliary cholangitis: a mendelian randomization study. Front Immunol. (2022) 13:1055953. doi: 10.3389/fimmu.2022.1055953, PMID: 36605198 PMC9807903

[36] GBD 2016 Healthcare Access and Quality Collaborators. Measuring performance on the healthcare access and quality index for 195 countries and territories and selected subnational locations: a systematic analysis from the global burden of disease study 2016. Lancet. (2018) 391:2236–71. doi: 10.1016/S0140-6736(18)30994-2, PMID: 29893224 PMC5986687

[37] National Health Commission of the People's Republic of China. Overview of China's healthcare quality and technical capacity development report - distributed by the National Healthcare Commission at its regular press conference on October 9. (2019). Available online at:http://www.nhc.gov.cn/xcs/s7847/201910/c3984f96380b4eb6bdbe5b8b4a399475.shtml [Accessed November 01, 2024].

[38] The Publicity Department of China. Increase in the level of health literacy to 29.70 per cent of the population nationwide by 2023. (2023). Available online at:http://www.nhc.gov.cn/xcs/s3582/202404/287e15ca9fd148b5ab9debce59f58c6d.shtml [Accessed November 01, 2024].

[39] Zhang Z Dong J Lin F Wang Q Xu Z He X. Hotspots and difficulties of biliary surgery in older patients. Chin Med J. (2023) 136:1037–46. doi: 10.1097/CM9.0000000000002589, PMID: 37052140 PMC10228479

[40] Gu Q Zhou G Xu T. Risk factors for gallstone disease in Shanghai: an observational study. Medicine (Baltimore). (2020) 99:e18754. doi: 10.1097/MD.0000000000018754, PMID: 32011459 PMC7220401

[41] Morgan AE Mooney KM Wilkinson SJ Pickles NA Mc Auley MT. Cholesterol metabolism: a review of how ageing disrupts the biological mechanisms responsible for its regulation. Ageing Res Rev. (2016) 27:108–24. doi: 10.1016/j.arr.2016.03.008, PMID: 27045039

[42] Sokal A Sauvanet A Fantin B de Lastours V. Acute cholangitis: diagnosis and management. J Visc Surg. (2019) 156:515–25. doi: 10.1016/j.jviscsurg.2019.05.007, PMID: 31248783

[43] Chong B Jayabaskaran J Kong G Chan YH Chin YH Goh R. Trends and predictions of malnutrition and obesity in 204 countries and territories: an analysis of the global burden of disease study 2019. EClinicalMedicine. (2023) 57:101850. doi: 10.1016/j.eclinm.2023.101850, PMID: 36864983 PMC9971264

[44] Zhou J Yu W Jiang G Li H Luo J Li S. Risk of gallstones increases with multiple dimensions of obesity indexes: a prospective study based on the UK biobank. Obes Facts. [Epub ahead of print] (2025):1–21. doi: 10.1159/000545346, PMID: 40139177 PMC12052360

[45] Ng M Fleming T Robinson M Thomson B Graetz N Margono C. Global, regional, and national prevalence of overweight and obesity in children and adults during 1980-2013: a systematic analysis for the global burden of disease study 2013. Lancet. (2014) 384:766–81. doi: 10.1016/S0140-6736(14)60460-8, PMID: 24880830 PMC4624264

[46] Tilman D Clark M. Global diets link environmental sustainability and human health. Nature. (2014) 515:518–22. doi: 10.1038/nature13959, PMID: 25383533

[47] Yang JL Huang JJ Cheng N Zhang S Liu SM Huang WY. Sex-specific and dose-response relationship between the incidence of gallstones and components of the metabolic syndrome in Jinchang cohort: a prospective study. Biomed Environ Sci. (2020) 33:633–8. doi: 10.3967/bes2020.084, PMID: 32933617

[48] Shabanzadeh DM Holmboe SA Sørensen LT Linneberg A Andersson AM Jørgensen T. Are incident gallstones associated to sex-dependent changes with age? A cohort study. Andrology. (2017) 5:931–8. doi: 10.1111/andr.12391, PMID: 28704597

[49] Stinton LM Shaffer EA. Epidemiology of gallbladder disease: cholelithiasis and cancer. Gut Liver. (2012) 6:172–87. doi: 10.5009/gnl.2012.6.2.172, PMID: 22570746 PMC3343155

[50] Ranelletti FO Piantelli M Farinon AM Zanella E Capelli A. Estrogen and progesterone receptors in the gallbladders from patients with gallstones. Hepatology. (1991) 14:608–12. doi: 10.1016/0270-9139(91)90046-x, PMID: 1916661

[51] Fielder S Nickkho-Amiry M Seif MW. Obesity and menstrual disorders. Best Pract Res Clin Obstet Gynaecol. (2023) 89:102343. doi: 10.1016/j.bpobgyn.2023.102343, PMID: 37279629

[52] Wang HH de Bari O Arnatt CK Liu M Portincasa P Wang DQ. Activation of estrogen receptor G protein-coupled receptor 30 enhances cholesterol cholelithogenesis in female mice. Hepatology. (2020) 72:2077–89. doi: 10.1002/hep.31212, PMID: 32112420 PMC8157628

[53] Baker P Machado P Santos T Sievert K Backholer K Russell C. Ultra-processed foods and the nutrition transition: Global, regional and national trends, food systems transformations and political economy drivers. Obes Rev. (2020) 21:e13126. doi: 10.1111/obr.13126 32761763

[54] Wang H Zhai F. Programme and policy options for preventing obesity in China. Obes Rev. (2013) 14:134–40. doi: 10.1111/obr.12106, PMID: 24102781 PMC4048452

[55] Beijing Tsinghua Changgung Hospital. (2019). China's Hepatobiliary Disease Standard Database Launched. Available online at:https://www.btch.edu.cn/xxdt/xwdt/53233.htm [Accessed April 14, 2025].

[56] Xiao W Huang X Wang JH Lin DR Zhu Y Chen C. Screening and identifying hepatobiliary diseases through deep learning using ocular images: a prospective, multicentre study. The Lancet Digital Health. (2021) 3:e88–97. doi: 10.1016/s2589-7500(20)30288-0, PMID: 33509389

[57] Shanghai Municipal Administrator of Traditional Chinese Medicine. (2024). Five “ones” to prevent gallstones. Available online at:https://wsjkw.sh.gov.cn/jthl/20240307/1e2bd1e9e47d444a919119acf6833d74.html [Accessed April 14, 2025].

[58] National Health Commission of the People’s Republic of China, etc. Circular on the issuance of a programme for the implementation of the “year of weight management” activities. (2024). Available online at:https://www.gov.cn/zhengce/zhengceku/202406/content_6959543.htm [Accessed April 14, 2025].

