# Supplementary material for: Burden and risk factors for gallbladder and biliary tract diseases in China from 1990 to 2021 and burden predictions of risk factors for the next 15 years
Source: Front Med (Lausanne). 2025 Aug 15;12:1528608. doi: 10.3389/fmed.2025.1528608 (PMC12394182; doi:10.3389/fmed.2025.1528608)
Supplement: Supplementary file 1 [file Data_Sheet_1.docx]

Supplementary Material


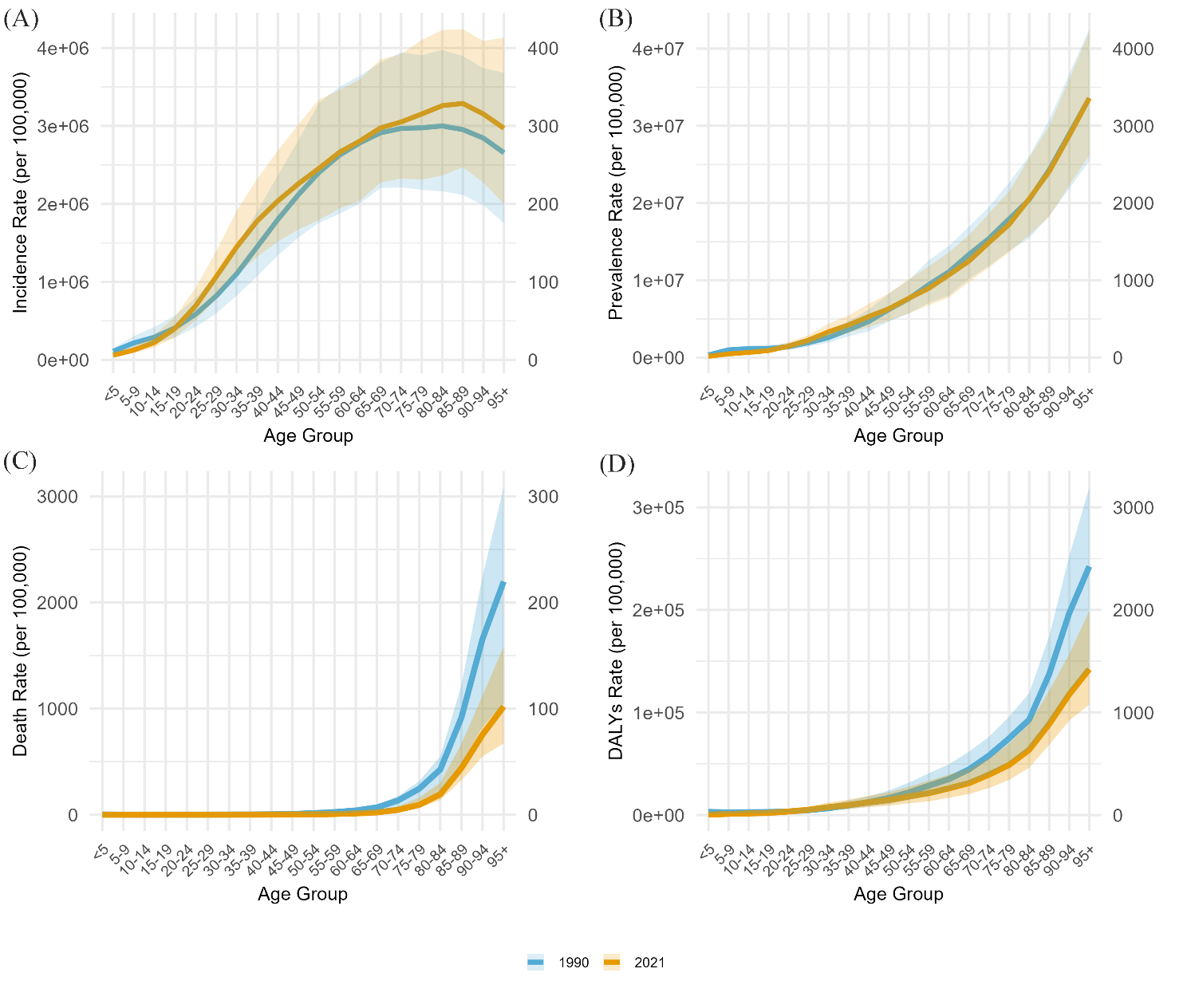


**Figure S1.** This figure compares the crude rates of incidence, prevalence, and DALYs across different age groups in China from 1990 to 2021. The following sections are included: (A) crude incidence rate; (B) crude prevalence rate; (C) crude mortality rate; and (D) crude DALY rate.


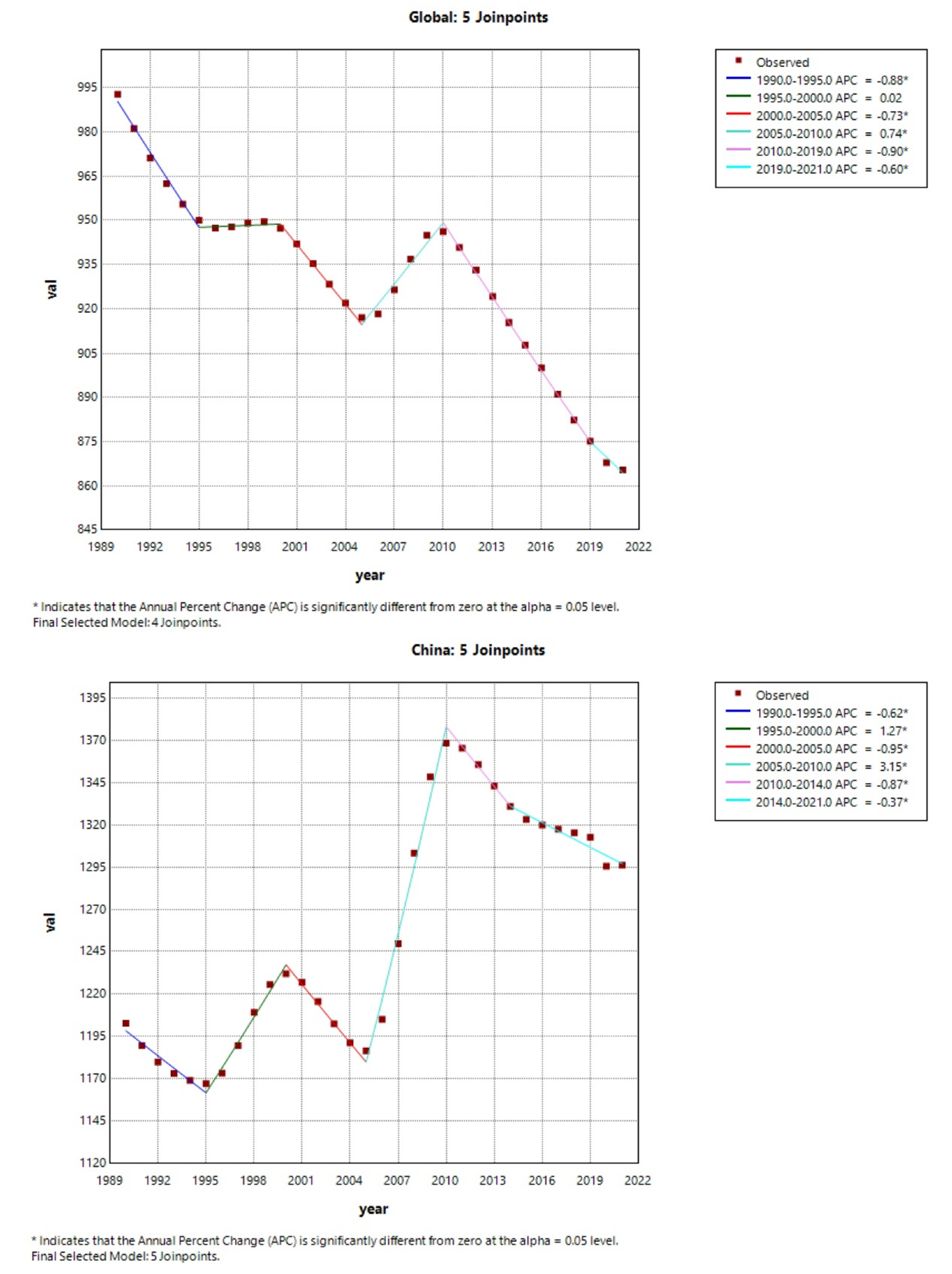


**Figure S2.** Joinpoint analysis of age-standardized incidence rate trends in China and globally.


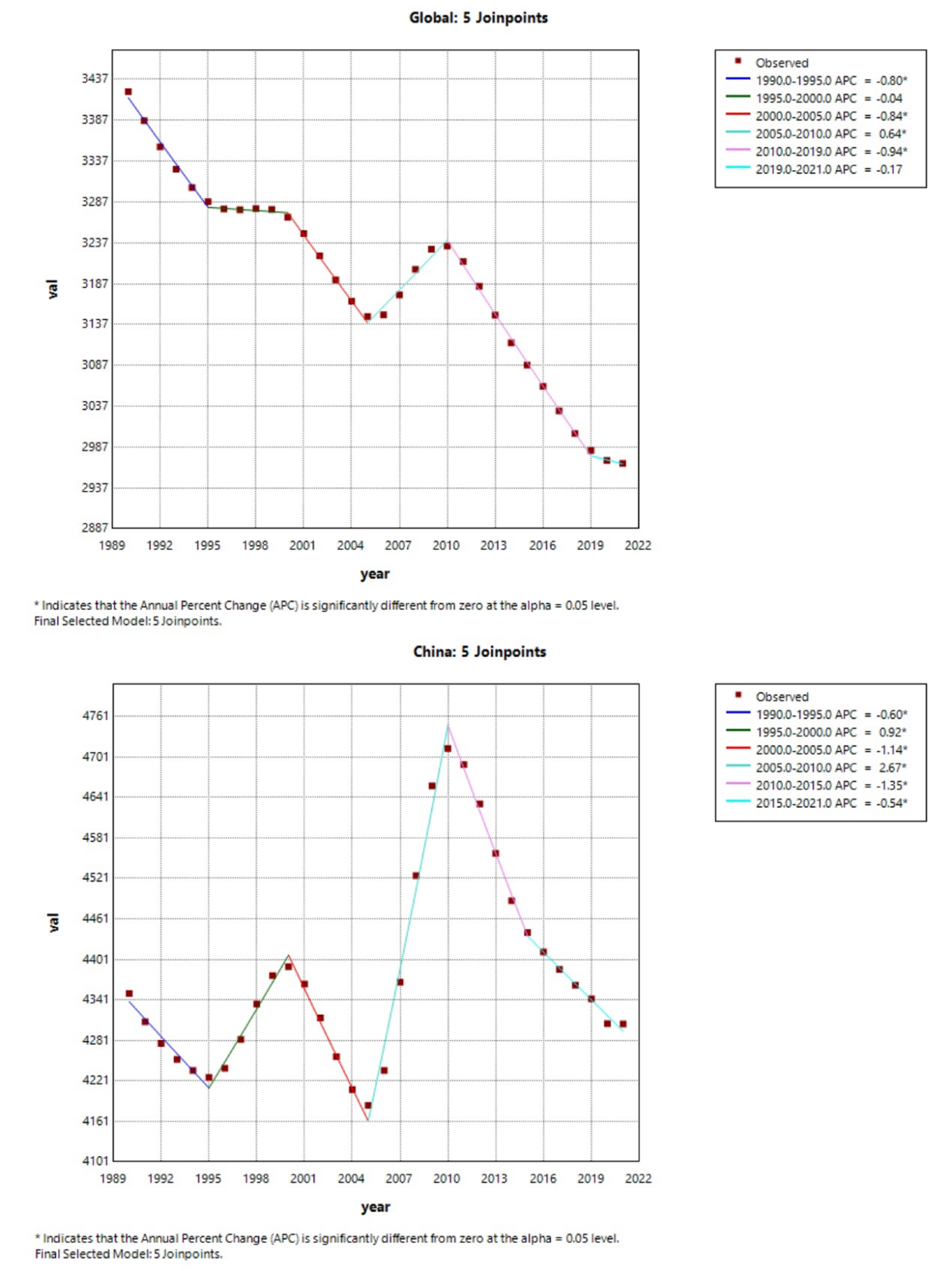


**Figure S3.** Joinpoint analysis of age-standardized prevalence rate trends in China and globally.

**
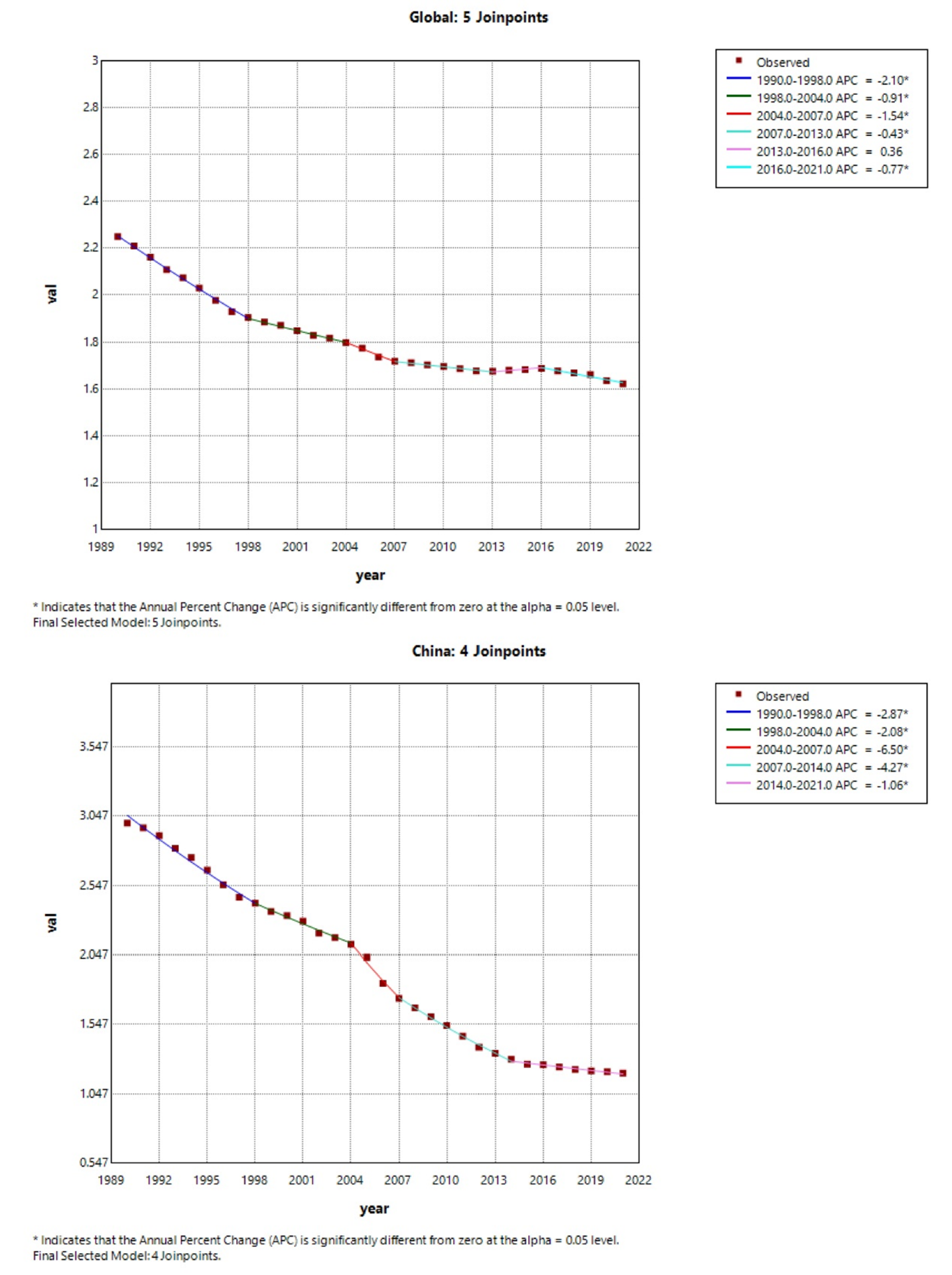
**

**Figure S4.** Joinpoint analysis of age-standardized deaths rate trends in China and globally.

**
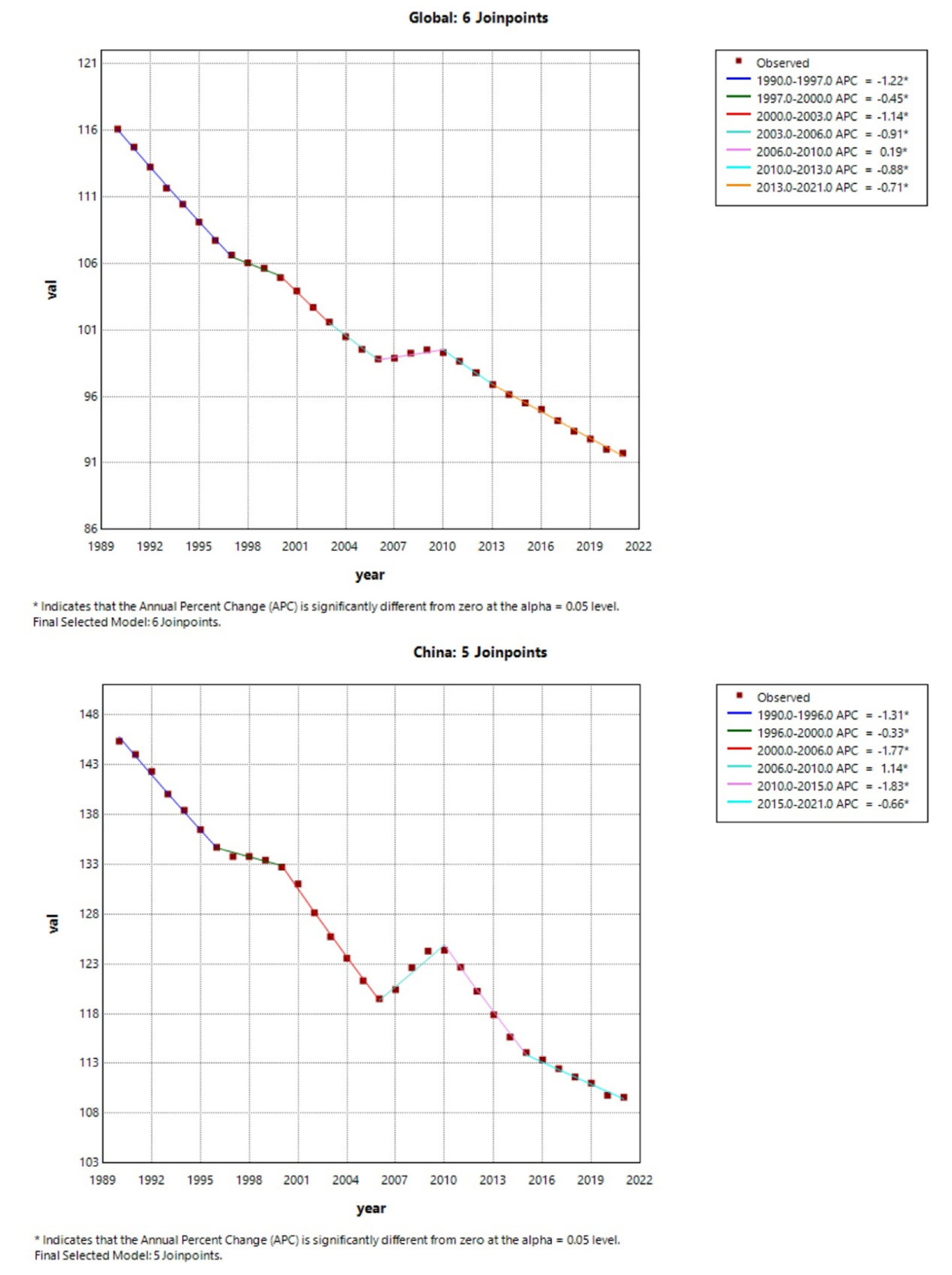
**

**Figure S5.** Joinpoint analysis of age-standardized DALYs trends in China and globally.


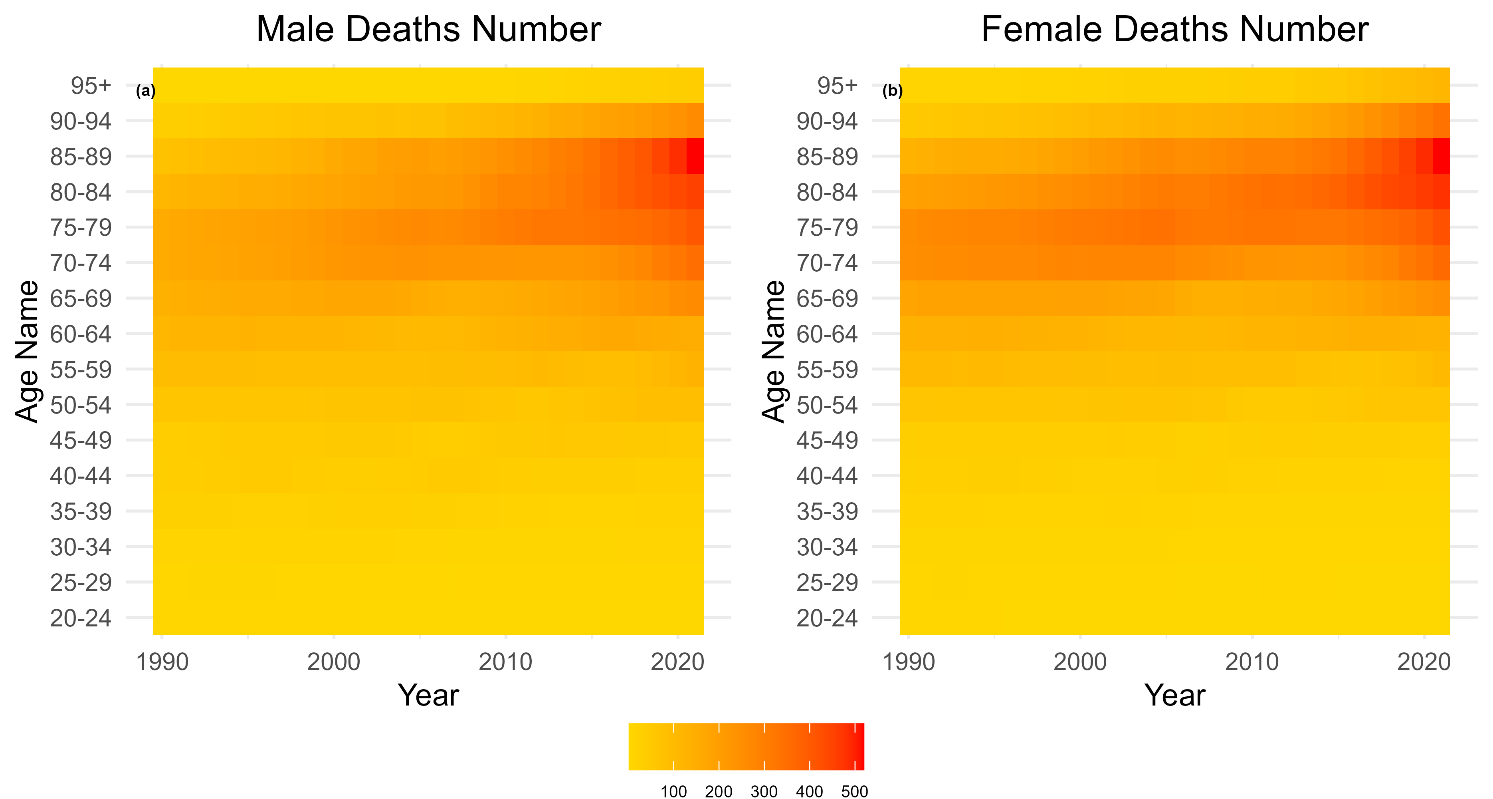


**Figure S6.** Distribution of deaths from GBDT attributed to HBMI by sex and age group from 1990-2021.


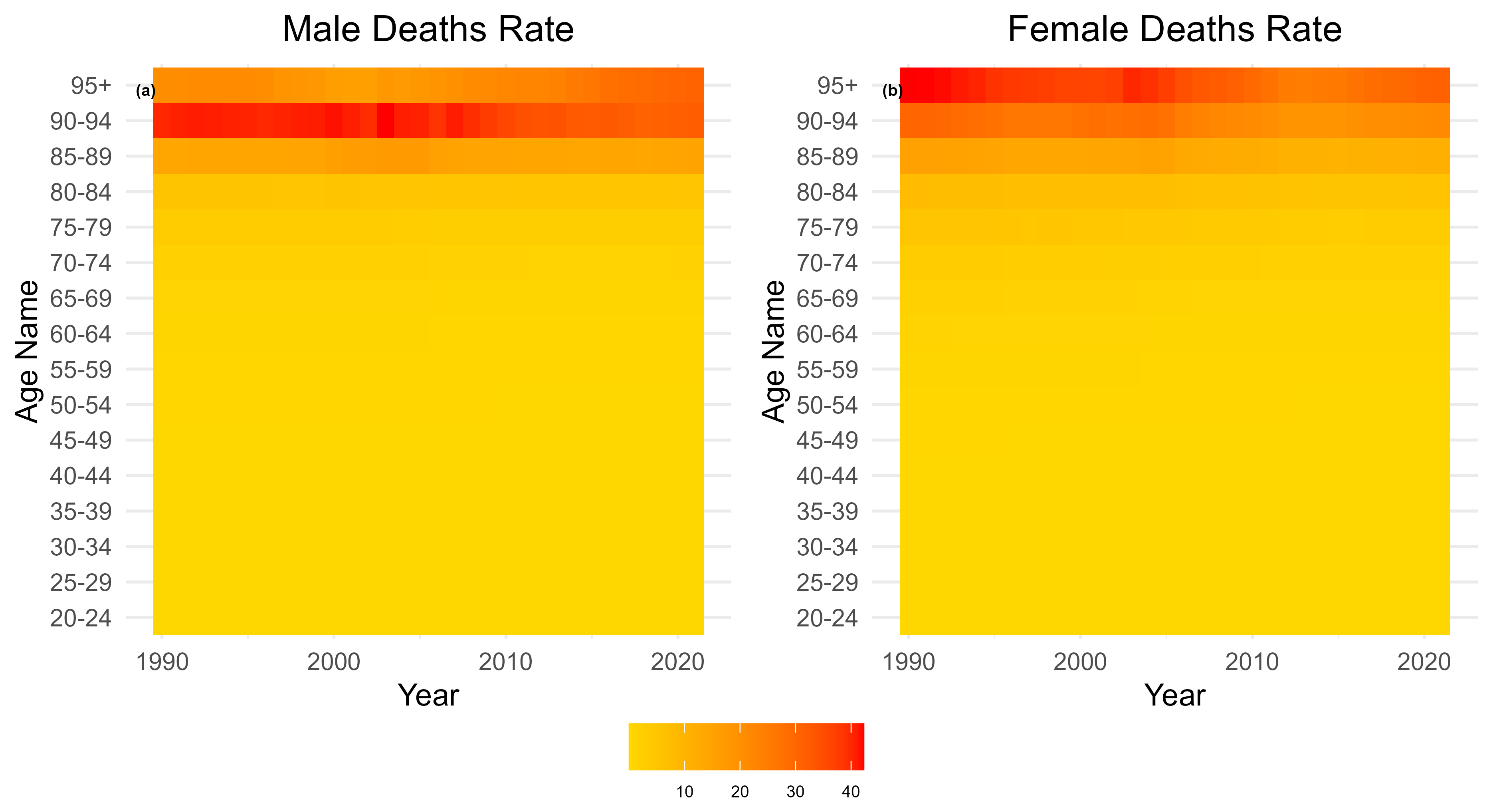


**Figure S7.** Distribution of death rate from GBDT attributed to HBMI by sex and age group from 1990-2021.


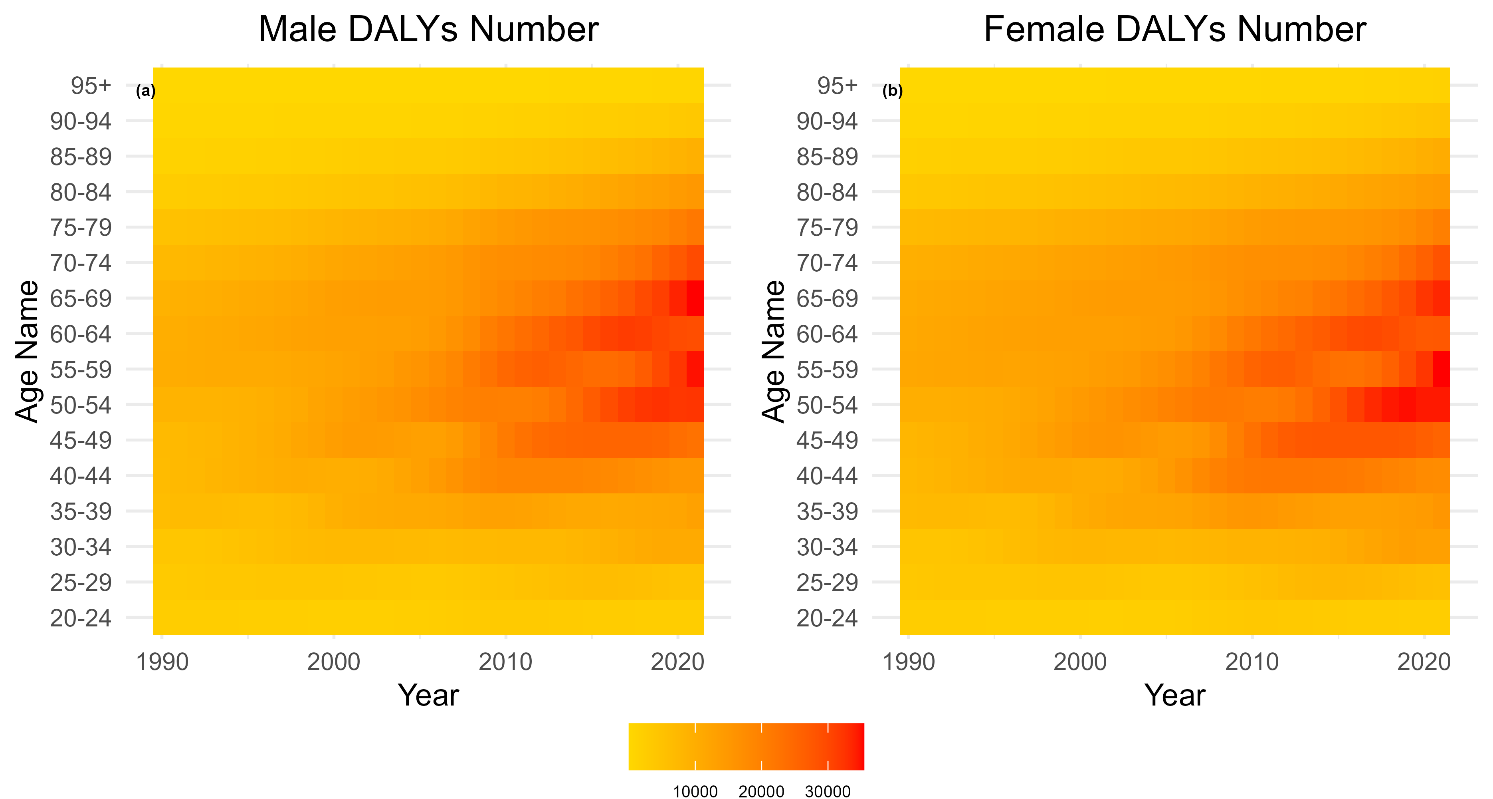


**Figure S8.** Distribution of DALYs from GBDT attributed to HBMI by sex and age group from 1990-2021.


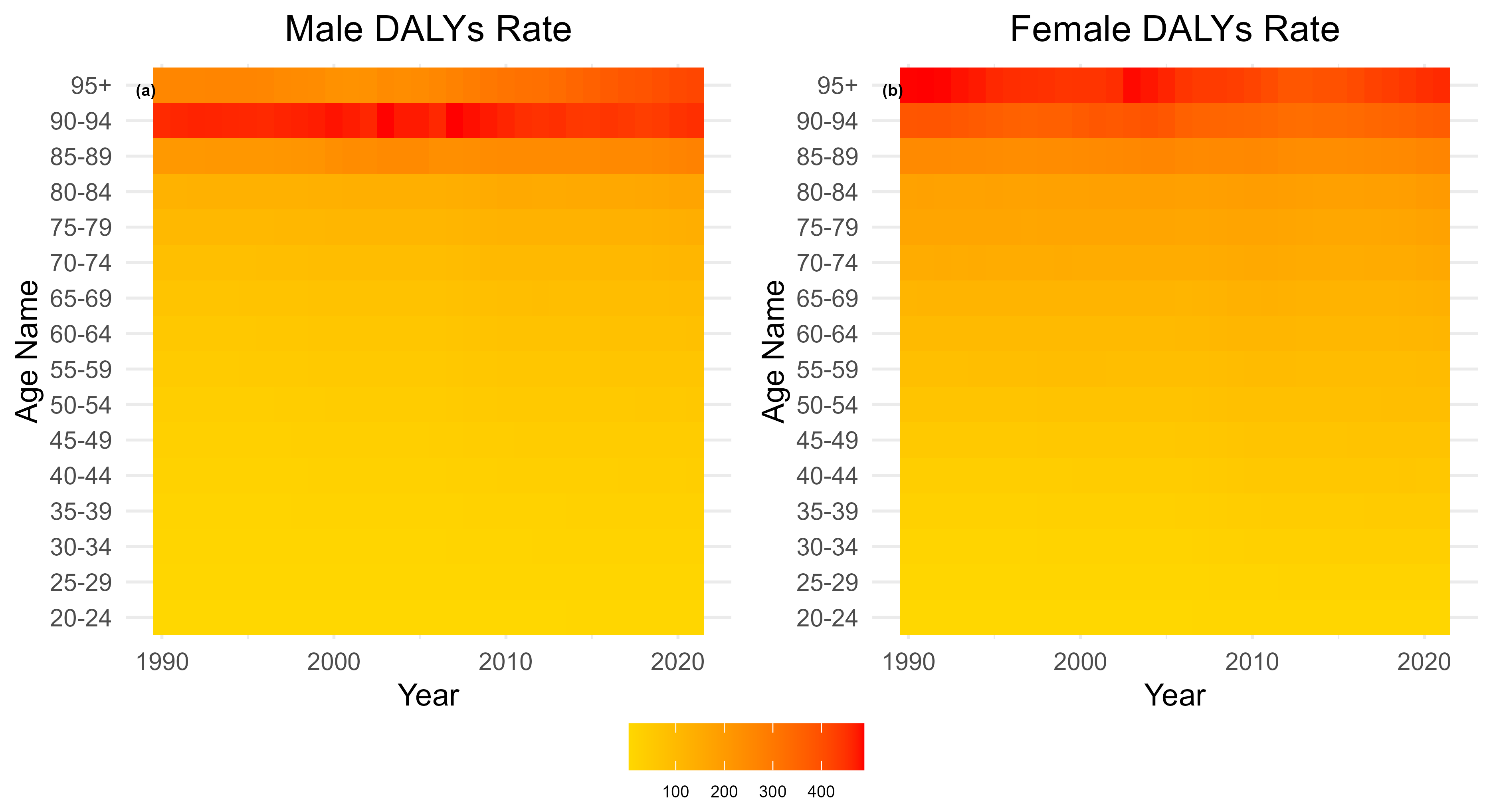


**Figure S9.** Distribution of DALYs rate for GBDT due to HBMI by sex and age group from 1990-2021.
